# The Versatility of Ethylene Glycol to Tune the Dimensionality and Magnetic Properties in Dy^III^-Anilato-Based Single-Ion Magnets

**DOI:** 10.1021/acs.cgd.2c01409

**Published:** 2023-01-17

**Authors:** Samia Benmansour, Cristina Pintado-Zaldo, Javier Martínez-Ponce, Antonio Hernández-Paredes, Antonio Valero-Martínez, Miriam Gómez-Benmansour, Carlos J. Gómez-García

**Affiliations:** Departamento de Química Inorgánica, Universidad de Valencia, Dr. Moliner 50, 46100Burjasot, Valencia, Spain

## Abstract

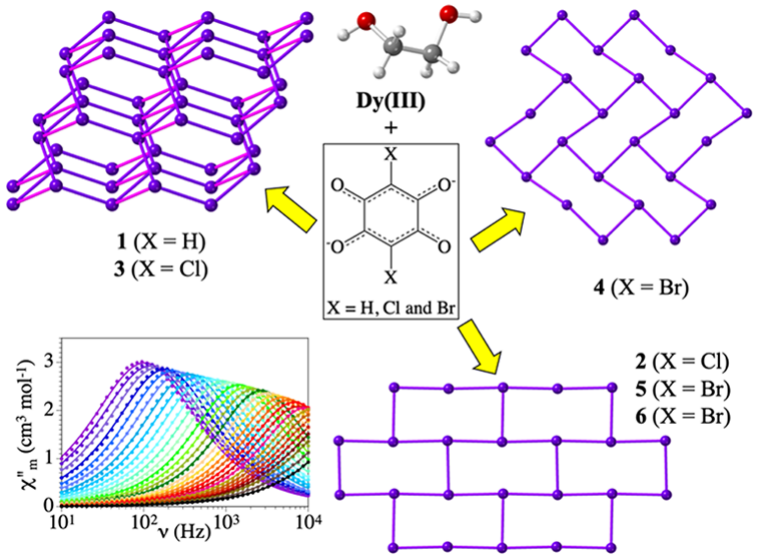

We exploit the high versatility of the solvent ethylene glycol (eg = CH_2_OH-CH_2_OH) acting as a ligand with three different coordination modes: terminal (κ*O*), chelate (κ^2^*O,O′*), and bridge (1κ*O*,2κ*O′*) to prepare a novel family of six different coordination polymers with Dy^III^ and three different anilato ligands (3,6-disubstituted-2,5-dihydroxy-1,4-benzoquinone dianion = C_6_O_4_X_2_^2–^, with X = H, Cl, and Br). With the X = H derivative (dhbq^2–^), we have prepared [Dy_2_(dhbq)_3_(eg)_2_(μ-eg)]·4eg·2H_2_O (**1**), a 3D diamond-like network with a chelate and bridging eg molecules. With the X = Cl derivative (chloranilato), we have prepared [Dy_2_(C_6_O_4_Cl_2_)_3_(eg)_4_]·2eg·H_2_O (**2**) and [Dy_2_(C_6_O_4_Cl_2_)_3_(μ-eg)(H_2_O)_4_]·2eg·7H_2_O (**3**). Compound **2** has a 2D (6,3)-gon brick-wall lattice and contains a chelate and a terminal eg molecule. Compound **3** has a 3D diamond-like topology as **1**, although now the chelate eg has been replaced by two water molecules. Finally, with the X = Br derivative (bromanilato), we have obtained [Dy_2_(C_6_O_4_Br_2_)_3_(eg)_2_(CH_3_OH)_2_]·2eg·4CH_3_OH (**4**), [Dy_2_(C_6_O_4_Br_2_)_3_(eg)_4_]·4eg (**5**), and [Dy_2_(C_6_O_4_Br_2_)_3_(eg)_3_(H_2_O)]·2eg·H_2_O (**6**). Compound **4** has a 2D (6,3)-gon herringbone topology and contains a chelate eg and a MeOH molecule. Compounds **5** and **6** have a 2D (6,3)-gon brick-wall topology with a chelate and a terminal eg molecules (in **5** and in one of the two independent Dy centers of **6**). The other Dy center in **6** has a chelate eg and a water molecule. All the compounds show slow relaxation of the magnetization at low temperatures (in compounds **1**, **2**, and **5** with no applied DC field). The magnetization of compounds **1**–**6** relaxes through Orbach and direct mechanisms when a DC field is applied and through an Orbach and/or quantum tunneling mechanism when no DC field is applied.

## Abstract 2

Reaction of Dy^III^ with three anilato derivative ligands and ethylene glycol gives rise to six different coordination polymers: two with a 3D diamond-like structure and four with 2D structures with (6,3) topology: three with a brick wall structure and one with a herringbone one. They all show slow relaxation of the magnetization: three with and three without the application of a DC field.

## Introduction

Two of the hottest topics in material science nowadays are (i) porous crystalline coordination polymers (CP), best known as metal organic frameworks (MOFs),^1,2^ and (ii) single-molecule (or single-ion) magnets (SMM and SIM).^3−7^ Combining these two topics is, therefore, a very appealing challenge, since MOFs behaving as SIM may find specific applications in different fields as magnetic-based sensors of different chemical species as gases, solvents, and contaminants.^8−10^ Although there are many examples of magnetic MOFs,^11−13^ very few show a slow relaxation of the magnetization, behaving as SMM or SIM.^14,15^ In most of these cases, the presence of a DC magnetic field is required to suppress the fast relaxation of the magnetization through a quantum tunneling mechanism (field-induced SMM or SIM).

A very promising and recent strategy to obtain MOFs with SIM or FI-SIM behavior consists of combining lanthanoid metals ions, mainly Dy^III^, with different bridging ligands to build extended structures. When the bridging ligands are poor magnetic couplers, the Ln(III) centers are well isolated (since the 4f orbitals are deep) and become good candidates to behave as SIM or FI-SIM. We have selected the Dy^III^ ion since it presents an oblate single-ion electron density and shows a strong axial crystal field below and above the equatorial plane, stabilizing the largest *m*_J_ and maximizing the uniaxial anisotropy.^16^

**Scheme 1:**
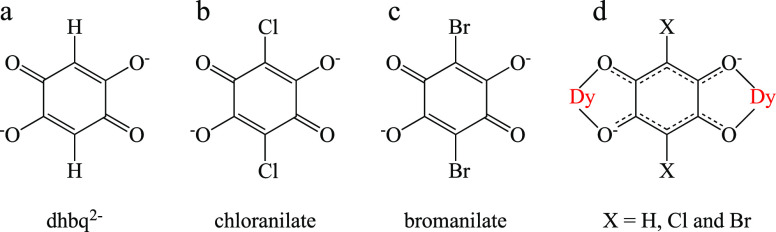
(a–c) Anilato Ligands Used in This Work and (d) Bis-bidentate Coordination Mode

As bridging ligands, we have selected anilato-type ligands (3,6-disubstituted-2,5-dihydroxy-1,4-benzoquinone dianion = C_6_O_4_X_2_^2–^, Scheme 1), as they are a large family of ligands (X = H, F, Cl, Br, I, NO_2_, Cl/CN, CH_3_, *t*-but, etc.)^17,18^ that can be used to construct two- and three-dimensional (2D and 3D) MOFs with different transition metal ions^19−21^ or lanthanoids.^22−37^

These studies show that anilato-type ligands usually coordinate in a bis-bidentate way (Scheme 1). Transition metal ions are usually hexacoordinated by three chelating anilato ligands, giving rise to 2D lattices with a honeycomb (6,3)-gon topology.^19−21^ In contrast, lanthanoids are usually octa- or nona-coordinated and appear surrounded by three (four in a few cases) chelating anilato ligands, the remaining coordination positions being occupied by solvent molecules as H_2_O, dimethylformamide (dmf), dimethyl-sulfoxide (dmso), etc. with a high coordination ability toward Ln(III) ions.^38,39^ The key role of these coordinated solvent molecules on structural aspects as the coordination number, geometry, and final structure, has been very recently pointed out by some of us.^26,27,29−33^

Despite the larger coordination number, when the Ln(III) ions are surrounded by three chelating anilato ligands, the most common topology is also the (6,3)-gon.^24^ Nevertheless, the presence of the solvent molecules produces a distortion of the 6-membered rings that appear as distorted hexagons^24,26,29,40,41^ or even as rectangles, giving rise to brick-wall^22,23,30,32,37^ and herringbone^26,28,29,31,37^ lattices. In the few cases where the Ln(III) ions are surrounded by four anilato ligands, the two most common topologies are the 2D (4,4)-gon square^27,36,42,43^ and the 3D (6,4)-gon diamond-like.^24,37^

The use of anilato ligands to construct these 2D and 3D lattices is based, among other properties, on their capacity to act as bis-bidentate bridges, connecting two metal atoms (Scheme 1d) and their capacity to magnetically isolate lanthanoid ions (as a result of the negligible overlap with the 4f orbitals). This last property has very recently started to be exploited to prepare MOFs with SIM and SMM behavior.^28,31−35,42^

Although the number of different solvent molecules used to complete the coordination sphere of the Ln(III) ions is quite high,^33^ all of them are rather simple monodentate molecules as H_2_O, ethanol, dimethylformamide (dmf = Me_2_NCHO), dimethyl sulfoxide (dmso = Me_2_SO), formamide (fma = NH_2_CHO), dimethylacetamide (dma = Me_2_NC(Me)O), and hexamethylphosphoramide (hmpa = (Me_2_N)_3_PO). To date, no attempt has been done to prepare anilato-based lattices with potentially bridging solvent molecules as ethylene glycol (eg = CH_2_OH-CH_2_OH). Here we show how the coordination versatility of eg may generate up to six different extended lattices when used with Dy^III^ and three different anilato ligands of the type (C_6_O_4_X_2_)^2–^ with X = H (dhbq^2–^), Cl (C_6_O_4_Cl_2_^2–^), and Br (C_6_O_4_Br_2_^2–^) (Scheme 1). Compound [Dy_2_(dhbq)_3_(eg)_2_(μ-eg)]·4eg·2H_2_O (**1**) is obtained with dhbq^2–^ and shows a 3D diamond-like lattice. With chloranilato (C_6_O_4_Cl_2_^2–^), using two slightly different syntheses, two different compounds have been obtained, formulated as [Dy_2_(C_6_O_4_Cl_2_)_3_(eg)_4_]·2eg·H_2_O (**2**) and [Dy_2_(C_6_O_4_Cl_2_)_3_(μ-eg)(H_2_O)_4_]·2eg·7H_2_O (**3**). Compound **2** is a 2D lattice with a brick-wall structure, whereas compound **3** shows a 3D diamond-like structure. Finally, using bromanilato (C_6_O_4_Br_2_^2–^), we have obtained three compounds using two different routes. These compounds are formulated as [Dy_2_(C_6_O_4_Br_2_)_3_(eg)_2_(CH_3_OH)_2_]·2eg·4CH_3_OH (**4**), with a 2D herringbone structure, [Dy_2_(C_6_O_4_Br_2_)_3_(eg)_4_]·4eg (**5**), with a 2D brick-wall structure, and [Dy_2_(C_6_O_4_Br_2_)_3_(eg)_3_(H_2_O)]·2eg·H_2_O (**6**), also with a 2D brick-wall structure. Herein, we show how slight changes in the synthetic conditions may lead to important changes in the structures and in the magnetic properties, thanks to the coordination versatility of the solvent ethylene glycol, that may act as bridging, chelate, and/or monodentate ligand.

### Experimental Section

Starting materials: All the chemicals and solvents were of reagent grade and used as received from commercial sources without further purification.

Given the limited number of crystals obtained in all cases, we checked the unit cell parameters of at least ten single crystals for each compound and verified that all the crystals used for the magnetic characterization for each compound had the same color and shape.

### Synthesis of [Dy_2_(dhbq)_3_(eg)_2_(μ-eg)]·4eg·2H_2_O (**1**)

Red plate-shape single crystals of compound **1** were obtained by carefully layering, at room temperature, a top solution of 2,5-dihydroxi-1,4-benzoquinone, H_4_C_6_O_4_ (2.8 mg, 20 μmol) in 5 mL of methanol, a middle phase (5 mL) of ethylene glycol, and a bottom solution of Dy(NO_3_)_3_·5H_2_O (8.8 mg, 20 μmol) in 5 mL of ethylene glycol. The tube was sealed and allowed to stand for about 3 weeks. Suitable crystals for X-ray diffraction were freshly picked and covered with paratone oil in order to avoid solvent loss to be characterized by single crystal X-ray diffraction.

### Synthesis of [Dy_2_(C_6_O_4_Cl_2_)_3_(eg)_4_]·2eg·H_2_O (**2**)

Violet block-shaped single crystals of compound **2** were obtained by carefully layering, at room temperature, a top solution of chloranilic acid, H_2_C_6_O_4_Cl_2_ (4.2 mg, 20 μmol) in 5 mL of methanol, a middle phase (5 mL) of ethylene glycol, and a bottom solution of Dy(NO_3_)_3_·5H_2_O (8.8 mg, 20 μmol) in 5 mL of ethylene glycol. The tube was sealed and allowed to stand for about 2 weeks. Suitable crystals for X-ray diffraction were freshly picked and covered with paratone oil in order to avoid solvent loss to be characterized by single crystal X-ray diffraction.

### Synthesis of [Dy_2_(C_6_O_4_Cl_2_)_3_(μ-eg)(H_2_O)_4_]·2eg·7H_2_O (**3**)

Violet plate-like single crystals of compound **3** were obtained by carefully layering, at room temperature, a top solution of chloranilic acid, H_2_C_6_O_4_Cl_2_ (4.2 mg, 20 μmol) in 5 mL of H_**2**_O:methanol (1:1), a middle phase (5 mL) of ethylene glycol, and a bottom solution of Dy(NO_3_)_3_·5H_2_O (8.8 mg, 20 μmol) in 5 mL of ethylene glycol. The tube was sealed and allowed to stand for about 1 week. Suitable crystals for X-ray diffraction were freshly picked and covered with paratone oil in order to avoid solvent loss to be characterized by single crystal X-ray diffraction.

### Synthesis of [Dy_2_(C_6_O_4_Br_2_)_3_(eg)_2_(CH_3_OH)_2_]·2eg·4CH_3_OH (**4**)

Violet hexagonal plate-like single crystals of compound **4** were obtained by carefully layering, at room temperature, a top solution of bromanilic acid, H_2_C_6_O_4_Br_2_ (6.0 mg, 20 μmol) in 5 mL of methanol, a middle phase (5 mL) of ethylene glycol, and a bottom solution of Dy(NO_3_)_3_·5H_2_O (8.8 mg, 20 μmol) in 5 mL of ethylene glycol. The tube was sealed and allowed to stand for 11 days. Suitable crystals for X-ray diffraction were freshly picked and covered with paratone oil in order to avoid solvent loss to be characterized by single crystal X-ray diffraction.

### Synthesis of [Dy_2_(C_6_O_4_Br_2_)_3_(eg)_4_]·4eg (**5**) and [Dy_2_(C_6_O_4_Br_2_)_3_(eg)_3_(H_2_O)]·2eg·H_2_O (**6**)

Pink plate-like single crystals of compound **5** were obtained by carefully layering, at room temperature, a top solution of bromanilic acid, H_2_C_6_O_4_Br_2_ (6.0 mg, 20 μmol) in 5 mL of H_2_O:methanol (1:1), a middle phase (5 mL) of ethylene glycol, and a bottom solution of Dy(NO_3_)_3_·5H_2_O (8.8 mg, 20 μmol) in 5 mL of ethylene glycol. The tube was sealed and allowed to stand for 1 week. Suitable crystals for X-ray diffraction were freshly picked and covered with paratone oil in order to avoid solvent loss to be characterized by single crystal X-ray diffraction.

When the previous solution is left undisturbed for more than 3 weeks, the pink plates become violet block-shaped single crystals of compound **6**. Suitable crystals for X-ray diffraction were freshly picked and covered with paratone oil to avoid solvent loss to be characterized by single crystal X-ray diffraction. Eight months later, in the previous tube, the crystals still have the same structure as compound **6**.

### Physical Measurements

Magnetic susceptibility of polycrystalline samples of complexes **1**–**6** was measured on a Quantum Design MPMS-XL-5 SQUID susceptometer with an applied magnetic field of 0.1 T in the temperature range 2–300 K. AC susceptibility measurements were performed on the same samples with an oscillating magnetic field of 0.8 mT in the frequency range 10–10000 Hz at low temperatures with different applied DC fields with a Quantum Design PPMS-9 equipment. The susceptibility data were corrected for the sample holder, and the corresponding diamagnetic contribution was evaluated using Pascal’s constants.^44^

### Crystallographic Data Collection and Refinement

Single crystals of compounds **1**–**6** were mounted on a mylar loop using a viscous hydrocarbon oil to coat the crystal and then transferred directly to the cold nitrogen stream for data collection. X-ray data were collected at 120 K on a Supernova diffractometer equipped with a graphite monochromated Enhance (Mo) X-ray Source (λ = 0.71073 Å). The program CrysAlisPro, Oxford Diffraction Ltd., was used for unit cell determinations and data reduction.^45^ Empirical absorption correction was performed using spherical harmonics, implemented in the SCALE3 ABSPACK scaling algorithm. Compound **1**, **3**, **5**, and **6** crystallize in the triclinic *P*1̅ space group, whereas compounds **2** and **4** crystallize in the monoclinic *P*2_1_*/n* and *P*2_1_*/c* space groups, respectively (Tables 1 and 2). Crystal structures were solved with the XT^46^ structure solution program using the Intrinsic Phasing solution method and by using Olex2^47^ as the graphical interface. The model was refined with version 2017/1 of XL^48^ using Least Squares minimization. All non-hydrogen atoms were refined anisotropically. Hydrogen atom positions were calculated geometrically and refined using the riding model. Most hydrogen atom positions were calculated geometrically and refined using the riding model, but some hydrogen atoms were refined freely.

**Table 1:** Crystal Data and Structure Refinement Parameters for Compounds **1**–**3**

complex	**1**	**2**	**3**
CCDC	2222691	2222692	2222693
formula	C_16_H_24_DyO_14_	C_13_H_12_Cl_3_DyO_10_	C_12_H_13_Cl_3_DyO_11_
formula weight	602.85	597.08	602.07
crystal system	triclinic	monoclinic	triclinic
space group	*P*1	*P*2_1_/*n*	*P*1
*a* (Å)	9.0589(13)	10.7543(2)	9.8035(5)
*b* (Å)	10.483(2)	16.7894(4)	9.9091(4)
*c* (Å)	12.506(2)	12.7262(3)	10.9070(6)
α (deg)	93.812(14)	90	88.054(4)
β (deg)	110.897(14)	95.396(2)	86.768(4)
γ (deg)	105.675(15)	90	72.351(4)
*V* (Å^3^)	1050.6(3)	2287.64(9)	1007.91(9)
*Z*	2	4	2
*ρ*_calc_(g cm^–3^)	1.906	1.734	1.984
μ (mm^–1^)	3.627	3.657	4.155
*R*_int_	0.0440	0.0305	0.0378
*T* (K)	120.00(14)	120.05(10)	119.6(8)
total refl.	6565	8242	7019
unique refl.	3701	4029	3547
reflections with *I* > 2(*I*)	3025	3337	3157
GOF^c^	1.093	1.026	1.059
*wR*_*2*_^b^	0.1825	0.0593	0.0900
*R*_1_ (all data)	0.0914	0.0378	0.0441
*R*_1_^a^	0.0730	0.0286	0.0378
largest peak/hole	3.89/–3.52	0.78/–0.62	2.31/–1.20

a *R*_1_ = **Σ**||*F*_0_| – |*F*_c_||/**Σ**|*F*_0_|.

b *wR*_2_ (*F*_0_^2^) = [Σ[*w*(*F*_0_^2^ – *F*_c_^2^)^2^/Σ*w F*_o_^4^]^1/2^.

c GOF = [Σ[*w*(*F*_0_^2^ – *F*_c_^2^)^2^/(*N*_obs_– *N*_params_)]^1/2^.

**Table 2:** Crystal Data and Structure Refinement Parameters for Compounds **4**–**6**

complex	**4**	**5**	**6**
CCDC	2222694	2222695	2222696
formula	C_16_H_23_Br_3_DyO_13_	C_17_H_23_Br_3_DyO_14_	C_28_H_34_Br_6_Dy_2_O_24_
formula weight	825.57	853.58	1559.01
crystal system	monoclinic	triclinic	triclinic
space group	*P*2_1_/*c*	*P*1	*P*1
*a* (Å)	10.3402(6)	10.7805(6)	10.1274(4)
*b* (Å)	20.4007(8)	10.7860(6)	12.6407(7)
*c* (Å)	12.6430(7)	12.4475(3)	17.1513(9)
α (deg)	90	96.366(3)	95.262(4)
β (deg)	113.524(7)	102.191(3)	106.145(4)
γ (deg)	90	113.955(5)	97.727(4)
*V* (Å^3^)	2445.4(2)	1261.17(1)	2070.61(18)
*Z*	4	2	2
*ρ*_calc_(g cm^–3^)	2.242	2.248	2.501
μ (mm^–1^)	8.019	7.781	9.458
*R*_int_	0.0518	0.0465	0.0352
*T* (K)	120.00(10)	120.00(10)	120.00(10)
total refl.	9338	8091	14483
unique refl.	4313	4431	7304
reflections with *I* > 2(*I*)	3512	3729	6006
GOF^c^	1.106	1.055	1.039
*wR*_2_^b^	0.2093	0.1394	0.0712
*R*_1_ (all data)	0.0940	0.0664	0.0482
*R*_1_^a^	0.0796	0.0552	0.0359
largest peak/hole	7.77/-1.98	3.234/-3.522	1.22/-0.96

a *R*_1_ = **Σ**||*F*_0_| – |*F*_c_||/**Σ**|*F*_0_|.

b *wR*_2_ (*F*_0_^2^) = [Σ[*w*(*F*_0_^2^ – *F*_c_^2^)^2^/Σ*w F*_o_^4^]^1/2^.

c GOF = [Σ[*w*(*F*_0_^2^ – *F*_c_^2^)^2^/(*N*_obs_– *N*_params_)]^1/2^.

A summary of the data collection and structure refinements for compounds **1**–**6** is given in Tables 1 and 2. CCDC 2222691–2222696 contain the supplementary crystallographic data for compounds **1**–**6**, respectively. Tables S1–S12 in the Supporting Information contain the bond distances and angles for compounds **1**–**6**.

## Results and Discussion

### Syntheses of the Compounds

The synthesis of compounds **1**–**6** was performed using a slow diffusion technique that allows the preparation of good quality single crystals. As can be seen in the Experimental Section and in Table 3, the synthetic conditions are the same for compounds **1**, **2**, and **4**. The only change is the anilato derivative (X = H in **1**, X = Cl in **2**, and X = Br in **4**). On the other side, if we change the MeOH solvent by a 1:1 mixture of H_2_O and MeOH in the top layer, we obtain compound **3** (for X = Cl) and compounds **5** and **6** (for X = Br). For X = H, the change of the solvent does not produce any change in the final product. It is to be noted that compounds **5** and **6** are prepared with exactly the same conditions and ligands (X = Br). The only difference is the crystallization time: compound **5** is obtained for short crystallization periods (1 week), whereas compound **6** is obtained by leaving the synthesis of **5** for longer crystallization times (between 3 weeks and at least eight months). We can, therefore, conclude that compound **6** is the thermodynamic phase, whereas **5** is the kinetic one. As we will discuss below, the differences between **5** and **6** are, on the one hand, the change of one monodentate eg molecule in **5** by a water molecule in half of the Dy^III^ ions in **6**. On the other hand, in **5** there is one independent Dy^III^ atom, while in **6** there are two crystallographically independent Dy^III^ centers.

**Table 3:** Synthetic Conditions and Structural Data of Compounds **1**–**6**

zone	reagents	**1** (H)	**2** (Cl)	**3** (Cl)	**4** (Br)	**5** (Br)	**6** (Br)
top	H_2_C_6_O_4_X_2_	20 μmol	20 μ mol	20 μ mol	20 μ mol	20 μ mol	20 μ mol
	solvent^a^	MeOH	MeOH	MeOH:H_2_O	MeOH	MeOH:H_2_O	MeOH:H_2_O
	volume	5 mL	5 mL	5 mL	5 mL	5 mL	5 mL
middle	solvent	eg	eg	eg	eg	eg	eg
bottom	Dy(NO_3_)_3_·5H_2_O	20 μmol	20 μmol	20 μmol	20 μmol	20 μmol	20 μmol
	solvent	eg	eg	eg	eg	eg	eg
	volume	5 mL	5 mL	5 mL	5 mL	5 mL	5 mL
time (weeks)		3	2	1	1.5	1	3
coordination environment of the Dy^III^ ions		chelate	chelate	bridge	chelate	chelate	chelate + terminal
		bridge	terminal	2H_2_O	MeOH	terminal	chelate + H_2_O
lattice		3D diamond	2D brick wall	3D diamond	2D herringbone	2D brick wall	2D brick wall

a All the solvent mixtures are 1:1 in volume; eg = ethylene glycol = CH_2_OH-CH_2_OH.

### Description of the Structures

#### Structure of [Dy_2_(dhbq)_3_(eg)_2_(μ-eg)]·4eg·2H_2_O (**1**)

**Figure 1:**
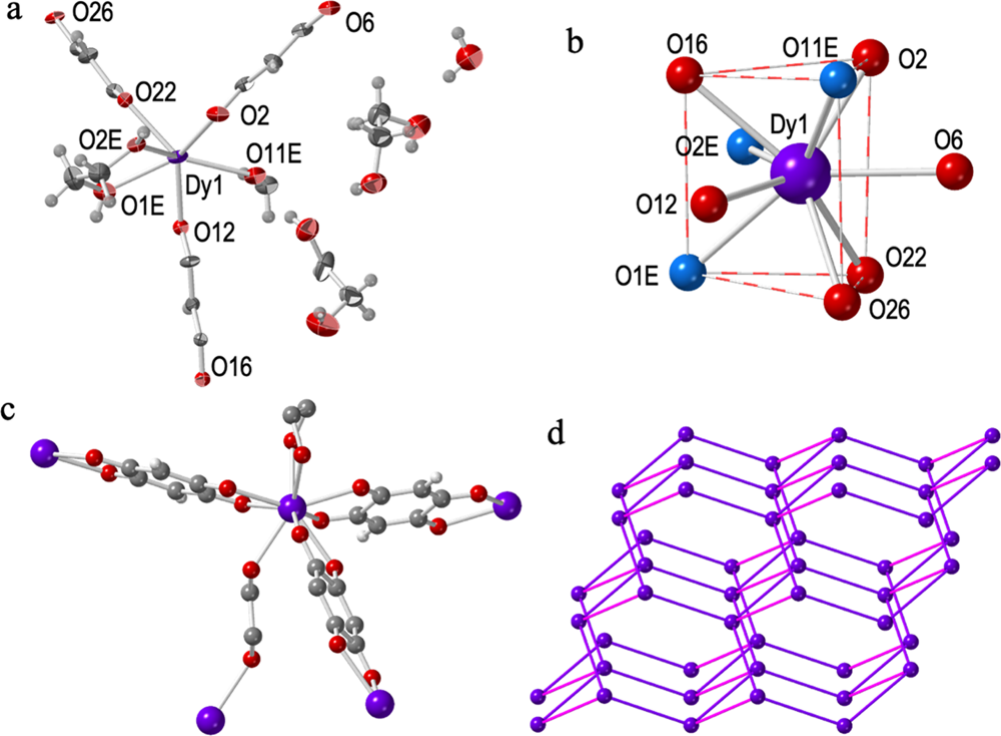
Structure of compound **1**: (a) Asymmetric unit with labeling scheme. Ellipsoids are drawn at 50% probability. (b) Tricapped trigonal prismatic coordination geometry of the Dy^III^ ion. O atoms from the ethylene glycol are displayed in blue. (c) Complete coordination environment around the Dy^III^ ions. H atoms of eg have been omitted for clarity. (d) 3D diamond-type structure. Purple and pink lines represent anilato and ethylene glycol bridges, respectively. Color code: Dy = purple, C = gray, O = red, and H = white.

Compound **1** crystallizes in the triclinic *P*1̅ space group (Table 1). Its asymmetric unit contains one Dy^III^ ion, half coordinated eg molecule, three halves dhbq^2–^ ligands, one chelating eg molecule, and one water and two eg crystallization molecules (Figure 1a). This gives a total formula of [Dy_2_(dhbq)_3_(eg)_2_(μ-eg)]·4eg·2H_2_O. Each Dy^III^ is nona-coordinated by six O atoms from three chelating anilato ligands, two O atoms from a chelating eg molecule, and one O atom from a bridging eg molecule. The analysis of the coordination environment of the Dy^III^ ion with the program SHAPE^49^ (Table 4) shows that it presents a distorted tricapped trigonal prismatic coordination geometry (Figure 1b). Each Dy^III^ ion is, therefore, connected to four other Dy^III^ ions in a distorted tetrahedral arrangement (through three bridging dhbq^2–^ ligands and one eg bridge, Figure 1c). This connectivity generates a 3D distorted diamond structure with a (6,4)-gon topology (Figure 1d).

**Table 4:** Continuous SHAPE Measurement (CShM) Values of the 13 Possible Coordination Geometries for the Dy^III^ Ion with Coordination Number Nine in Compounds **1**–**6**^a^

geometry	symmetry	**1**	**2**	**3**	**4**	**5**	**6** (Dy1)	**6** (Dy2)
EP-9	D9h	36.704	37.728	36.510	35.902	36.831	36.446	37.444
OPY-9	C8v	21.360	21.433	22.108	21.907	21.520	21.256	21.035
HBPY-9	D7h	20.061	19.187	17.056	18.903	19.373	18.729	18.205
JTC-9	C3v	15.639	15.374	16.604	16.164	15.562	16.048	16.778
JCCU-9	C4v	10.671	9.772	8.916	9.567	10.365	9.162	9.263
CCU-9	C4v	9.421	8.498	7.651	8.340	9.355	7.635	7.832
JCSAPR-9	C4v	2.001	2.267	2.121	1.864	1.694	1.795	2.151
**CSAPR-9**	**C4v**	0.927	0.966	1.165	0.935	**0.513**	**0.617**	**1.102**
JTCTPR-9	D3h	2.301	2.504	2.337	2.479	2.287	3.220	3.014
**TCTPR-9**	**D3h**	**0.562**	**0.508**	**0.695**	**0.860**	0.779	1.481	1.212
JTDIC-9	C3v	12.299	10.841	12.545	12.426	11.874	12.135	11.868
HH-9	C2v	11.250	11.666	10.467	10.812	11.723	11.327	9.570
MFF-9	Cs	1.396	1.505	1.554	1.279	1.253	0.947	1.288

a The minimum values are indicated in bold. EP-9 = Enneagon; OPY-9 = Octagonal pyramid; HBPY-9 = Heptagonal bipyramid; JTC-9 = Triangular cupola (J3) = trivacant cuboctahedron; JCCU-9 = Capped cube (Elongated square pyramid, J8); CCU-9 = Capped cube; JCSAPR-9 = Capped square antiprism (Gyroelongated square pyramid J10); **CSAPR-9 = Capped square antiprism**; JTCTPR-9 = Tricapped trigonal prism (J51); **TCTPR-9 = Tricapped trigonal prism**; JTDIC-9 = Tridiminished icosahedron (J63); HH-9 = Hula-hoop; MFF-9 = Muffin.

The Dy–O bond distances are similar to those found in other related structures with nona-coordinated Dy^III^ and anilato ligands.^27,30,32,34,36^ The average Dy–O_dhbq_ bond distances (2.387 Å) are slightly shorter than the Dy–O_eg_ ones (2.446 Å, Table S1, Supporting Information). This fact contrasts with the general trend observed for other similar compounds with chloranilato and bromanilato ligands and simple solvents,^27,30,32,34,36^ and can be attributed to two effects: (i) the higher electron density on the O atoms in the dhbq^2–^ ligand due to the lower electronegativity of H compared with Cl and Br. (ii) The tension in the five membered chelate ring for ethylene glycol and the bridging nature of the other coordinated eg molecule.

#### Structure of [Dy_2_(C_6_O_4_Cl_2_)_3_(eg)_4_]·2eg·H_2_O (**2**)

**Figure 2:**
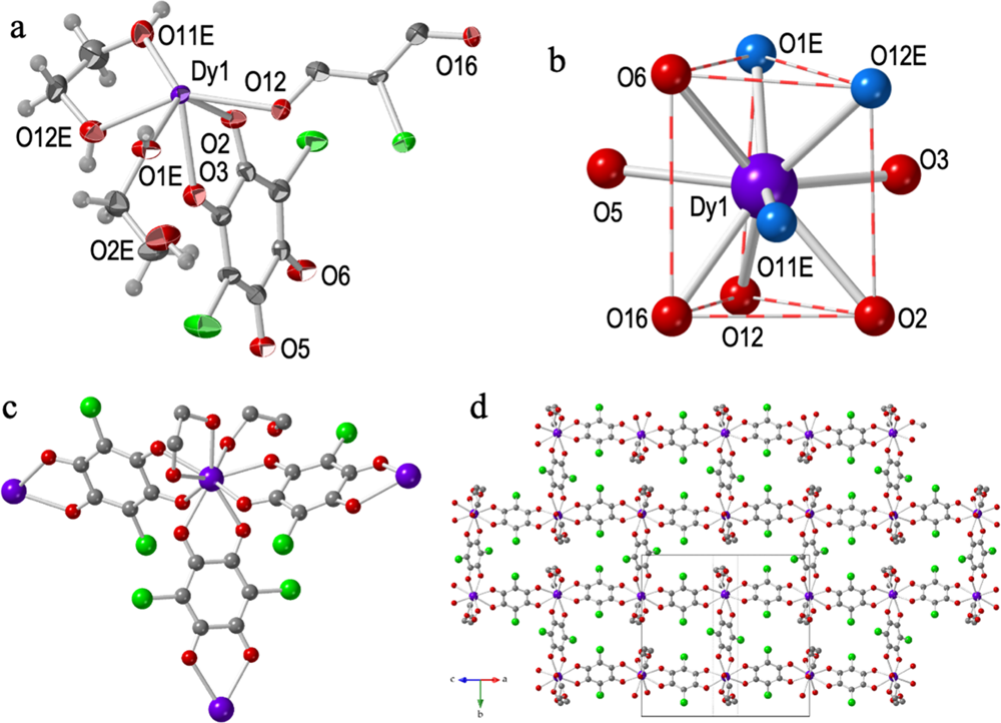
Structure of compound **2**: (a) Asymmetric unit with labeling scheme. Ellipsoids are drawn at 80% probability. (b) Tricapped trigonal prismatic coordination geometry of the Dy^III^ ion. O atoms from the ethylene glycol are displayed in blue. (c) Complete coordination environment around the Dy^III^ ions. H atoms have been omitted for clarity. (d) View of one layer showing the 2D brick-wall type structure. Color code: Dy = purple, C = gray, O = red, and Cl = green.

Compound **2** crystallizes in the monoclinic *P*2_1_*/n* space group (Table 1). Its asymmetric unit contains one Dy^III^ ion, one and half dhbq^2–^ ligands, and one terminal and one chelate coordinated eg molecules (Figure 2a). There are also one disordered eg and half water molecule as determined with the solvent mask command (see Supporting Information). The total formula is, therefore, [Dy_2_(C_6_O_4_Cl_2_)_3_(eg)_4_]·2eg·H_2_O. As in **1**, each Dy^III^ is nona-coordinated by six O atoms from three chelating anilato ligands, two O atoms from a chelating eg molecule, and one O atom from a terminal eg molecule in a distorted tricapped trigonal prismatic coordination geometry (Figure 2b), as shown by the analysis with the program SHAPE^49^ (Table 4). In **2**, each Dy^III^ ion is, therefore, connected to three other Dy^III^ ions through three bridging chloranilato ligands (Figure 2c) and forms rectangular rings (Figure S1) with six Dy^III^ centers in a (6,3)-gon topology. This connectivity generates layers parallel to the (101) plane (Figure S1) with a brick wall structure (Figure 1d). The layers are formed by rectangular cavities packed in an eclipsed way generating rectangular channels along the *a* direction (Figure S1).

The Dy–O bond distances are also similar to those found in other related structures with nona-coordinated Dy^III^ and anilato ligands.^27,30,32,34,36^ As observed in **1**, the average Dy–O_anilato_ bond distances (2.408 Å) are slightly shorter than the Dy–O_eg_ ones (2.428 Å, Table S3), although now the difference is shorter than in **1**. This fact is due to the longer average Dy–O_anilato_ bond distance observed for chloranilato as a consequence of the lower electron density in the O atoms of the chloranilato ligand.

#### Structure of [Dy_2_(C_6_O_4_Cl_2_)_3_(μ-eg)(H_2_O)_4_]·2eg·7H_2_O (**3**)

Compound **3** crystallizes in the triclinic *P*1̅ space group (Table 1). Its structure is very similar to that of compound **1**. Besides the obvious change in the ligand (dhbq^2–^ in **1***vs* chloranilato in **3**), the only differences are the presence of two coordinated water molecules in **3**, instead of a chelate eg molecule in **1** and the crystallization solvent molecules (four eg and two H_2_O molecules in **1***vs* two eg and seven H_2_O molecules in **3**). The asymmetric unit of **3** contains one Dy^III^ ion, half coordinated eg molecule, three halves chloranilato ligands, and two coordinated water molecules. There are also three and a half water and one eg crystallization molecules (Figure S2). The coordination number and geometry (Table 4), the connectivity, and the topology (diamond like) are the same in **1** and **3** (Figure S2). As expected, the average Dy–O_anilato_ bond distances are slightly longer in **3** (2.413 Å, Table S5) than in **1** (2.387 Å) since **3** contains chloranilato instead of dhbq^2–^.

#### Structure of [Dy_2_(C_6_O_4_Br_2_)_3_(eg)_2_(CH_3_OH)_2_]·2eg·4CH_3_OH (**4**)

**Figure 3:**
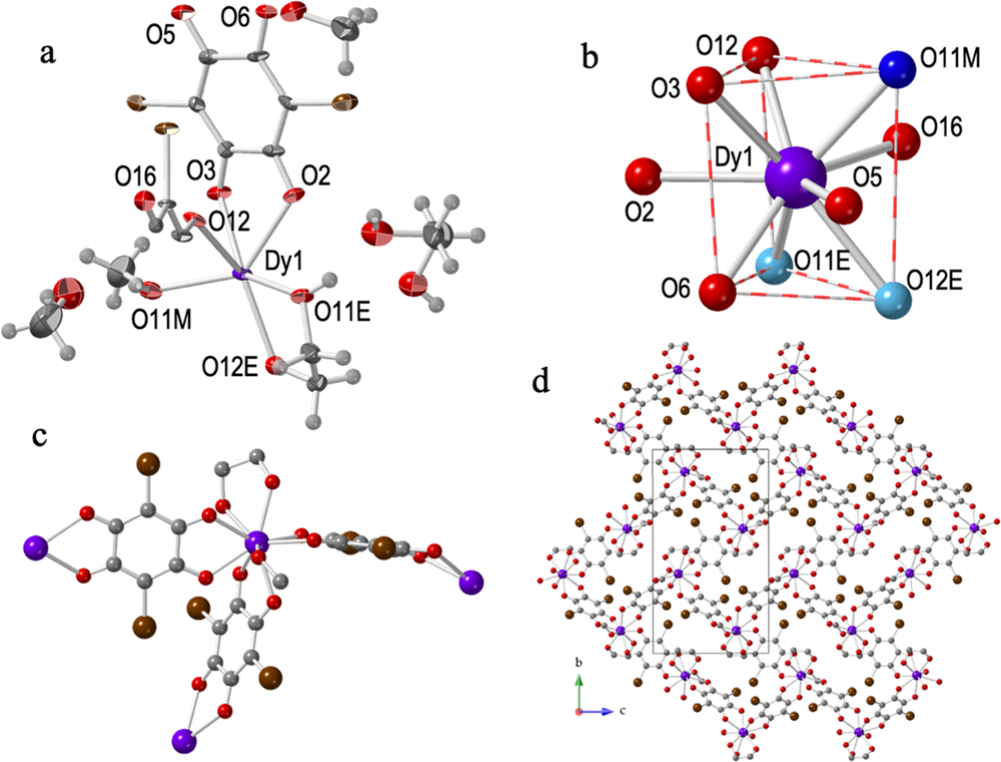
Structure of compound **4**: (a) Asymmetric unit with labeling scheme. Ellipsoids are drawn at 50% probability. (b) Tricapped trigonal prismatic coordination geometry of the Dy^III^ ion. O atoms from methanol and ethylene glycol are displayed in dark and light blue, respectively. (c) Complete coordination environment around the Dy^III^ ions. H atoms have been omitted for clarity. (d) View of one layer showing the 2D herringbone type structure. Color code: Dy = purple, C = gray, O = red, H = white, and Br = brown.

Compound **4** crystallizes in the monoclinic *P*2_1_*/c* space group (Table 2). Its asymmetric unit contains one Dy^III^ ion, one and half bromanilato ligands, one coordinated methanol molecule, and one chelate coordinated eg molecule (Figure 3a). There are also one eg and two methanol crystallization molecules. The total formula is, therefore, [Dy_2_(C_6_O_4_Br_2_)_3_(eg)_2_(MeOH)_2_]·2eg·4MeOH. Compound **4** is very similar to compound **2** and shows the same coordination geometry (Figure 3b) and connectivity (Figure 3c). Nevertheless, besides the change in the anilato ligand (bromanilato in **4***vs* chloranilato in **2**), there are some important differences: (i) the presence of a coordinated methanol molecule in **4** replacing a terminal eg in **2**, (ii) the crystallization solvent molecules, and (iii) the relative position of the coordinated anilato ligands in the coordination environment (Figure 3b). Thus, in **4** the two oxygen atoms of the three chelate anilato ligands occupy one coordination position in the equatorial plane and the other in one of the two triangular faces (eq-tr). This spatial orientation of the bromanilato ligands also results in planar layers (Figure S3) with rectangular cavities (as in **2**), but now the rectangles show a different relative orientation, giving rise to a herringbone structure in the layers (Figure 3d). Note that in compound **2**, only two of the three anilato ligands occupy one position in the equatorial plane and the other in one triangular face (eq-tr). The third anilato (O12/O16) occupies two positions in one triangular face (tr-tr) (Figure 2b). As in **2**, the layers are packed eclipsed along the *a* direction, giving rise to rectangular channels (that contain the crystallization methanol molecules, Figure S3). The average Dy–O_anilato_ bond distances are similar in **4** (2.409 Å) and **2** (2.408 Å). This unexpected fact (they should be shorter in the bromanilato derivative, **4**) can be attributed to the larger steric effect caused by the chelate eg and the MeOH molecules in **4**, since the average Dy–O_eg_ and Dy–O_MeOH_ in **4** (2.413 Å, Table S7) are shorter than the corresponding Dy–O_eg_ distances in **2** (2.428 Å).

#### Structure of [Dy_2_(C_6_O_4_Br_2_)_3_(eg)_4_]·4eg (**5**)

Compound **5** crystallizes in the triclinic *P*1̅ space group (Table 2). Its asymmetric unit contains one Dy^III^ ion, three-halves bromanilato ligands, and one chelate and one terminal coordinated eg molecules (Figure S4). There are also two eg crystallization molecules. The total formula is, therefore, [Dy_2_(C_6_O_4_Br_2_)_3_(eg)_4_]·4eg. Compound **5** is very similar to compound **2**. In fact, besides the change of chloranilato in **2** by bromanilato in **5**, the only difference is the coordination geometry (distorted tricapped trigonal prims in **2***vs* distorted capped square antiprism in **5**, Table 4 and Figure S4). Despite this difference, the connectivity, the shape of the cavities (rectangles, Figure S5), and their disposition (brick-wall, Figure S4) are the same in both compounds. The layers are also packed in an eclipsed way forming channels parallel to the *a* direction (Figure S5). The average Dy–O_anilato_ and Dy–O_eg_ bond distances in **5** (2.399 and 2.415 Å, respectively, Table S9) are also very similar to those observed in **2** (2.408 and 2.428 Å, respectively).

#### Structure of [Dy_2_(C_6_O_4_Br_2_)_3_(eg)_3_(H_2_O)]·2eg·H_2_O (**6**)

**Figure 4:**
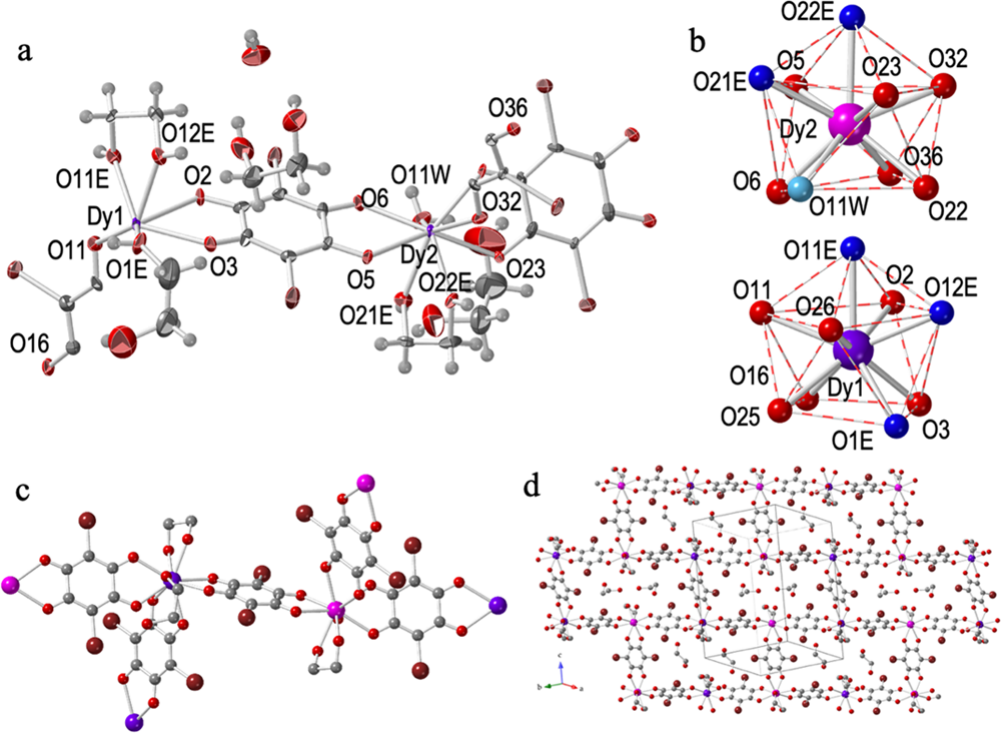
Structure of compound **6**: (a) Asymmetric unit with labeling scheme. Ellipsoids are drawn at 80% probability. (b) Tricapped trigonal prismatic coordination geometry of the Dy^III^ ions. O atoms from the ethylene glycol and water molecule are displayed in dark and light blue, respectively. (c) Complete coordination environment around the Dy^III^ ions. H atoms have been omitted for clarity. (d) View of one layer showing the 2D brick-wall type structure. Color code: Dy = purple, C = gray, O = red, and Cl = green.

Compound **6** crystallizes in the triclinic *P*1̅ space group (Table 2). Its asymmetric unit contains two Dy^III^ ions, two complete and two half bromanilato ligands, two chelate and one terminal coordinated eg molecules, and one coordinated water molecule (Figure 4). There are also two eg and one water crystallization molecules. The total formula is, therefore, [Dy_2_(C_6_O_4_Br_2_)_3_(eg)_3_(H_2_O)]·2eg·H_2_O. The structure of compound **6** is similar to **2** and **5**, although there are some slight differences. In **6** three are two independent Dy^III^ centers. Dy1 is surrounded by three bromanilato ligands one chelate and one terminal eg molecule. Dy2 has a coordinated water molecule instead of the terminal eg molecule (Figure 4a). Both Dy^III^ centers show a capped square antiprism geometry (Figure 4b), as observed in **5**. Each Dy1 center is connected, through three bromanilato bridges, to two Dy2 and one Dy1 centers, whereas each Dy2 is connected to two Dy1 and one Dy2 (Figure 4c). Compound **6** also forms parallel layers with rectangular rings (Figure S6) with a brick-wall structure (Figure 4d). As in **5**, the layers are packed in an eclipsed way, leading to rectangular channels along the *a* direction (Figure S6). The average Dy–O_anilato_ bond distances in **6** (2.410 Å for Dy1 and 2.393 Å for Dy2, Table S11) are similar to the Dy–O_eg_ ones (2.410 Å for Dy1 and 2.411 Å for Dy2) and shorter than the Dy–O_w_ in Dy2 (2.447 Å). As expected, the average Dy–O_bromanilato_ bond distances are very similar to those found in compounds **4** (2.409 Å) and **5** (2.399 Å).

As mentioned above, compound **5** transforms into compound **6** after a few weeks inside the tube. As a consequence, a new structure was obtained with two crystallographically independent Dy^III^ atoms. In half of them, one terminal eg molecule is replaced by a water molecule. This change is assisted by a shortening of the chelate Dy–O_eg_ bond distances (from 2.419 Å in **5** to 2.392 and 2.411 Å in **6**) and by a lengthening of the terminal Dy–O_eg_ bond distance (from 2.408 Å in **5** to 2.442 in **6**).

#### Role of the Solvent Coordination Mode on the Structure of Compounds **1**–**6**

The analysis of the structures of compounds **1**–**6**, shows different conclusions: (i) The two 3D structures (compounds **1** and **3**) present a bridging eg molecule that constitutes the fourth connection to obtain the diamond-like structure. In compound **1**, there is a chelate eg molecule occupying the two remaining coordination positions, whereas in **3**, these positions are occupied by two water molecules, probably due to a reduction in the free space when passing from X = H (in **1**) to X = Cl (in **3**). (ii) Compounds **2**, **5**, and **6** show the same brick-wall 2D topology and also the same coordination environment in the Dy centers (one chelate and one terminal eg molecule). In these three compounds, the chelate eg molecule reduces the available space and only allows the coordination of a terminal eg molecule (or a H_2_O molecule in one of the two Dy atoms in **6**). (iii) Compound **4**, although it also has a chelate eg molecule, is the only one with a herringbone structure topology. This original topology is due to the different disposition of the chelate eg molecule. Thus, in **4**, the chelate eg molecule occupies two positions in the same triangular face with the methanol molecule in the other triangular face, leading to the formation of a herringbone structure. This disposition contrasts with that in the brick-wall structures (**2**, **5**, and **6**), where the chelate eg occupies a position in the equatorial plane and a position in one triangular face, with the extra molecule in the same triangular phase. In summary, the bridging coordination mode of eg leads to 3D structures with a diamond-like topology, whereas the chelating coordination mode of eg plus a monodentate ligand leads to 2D structures with the 6,3-gon topology.

#### Magnetic Properties

The magnetic properties of compounds **1**–**6** are, in general, quite similar, although there are important differences due to the presence of different coordination environments. The product of the molar magnetic susceptibility per formula unit (two Dy^III^ ions) times the temperature (χ_m_T) shows in all cases a room temperature value of 28.2–28.7 cm^3^ K mol^–1^ (Figure S7). This value is close to the expected one for two independent Dy^III^ ions (28.34 cm^3^ K mol^–1^),^50^ in agreement with the good magnetic isolation provided by the anilato ligands when connecting Ln(III) ions.^33^ When the temperature is decreased, χ_m_*T* remains almost constant down to ca. 100 K and below this temperature, it shows a progressive decrease attributed to the depopulation of the excited levels that appear due to the ligand field (Figure S7).^50^ This good magnetic isolation and the behavior observed in other Dy^III^-anilato lattices^28,31,32,34^ prompted us to perform AC susceptibility measurements to check a possible single-ion magnet (SIM) or field-induced SIM behavior in compounds **1**–**6**.

When no DC field is applied, the AC susceptibility measurements show, in compounds **1**, **2**, and **5**, the presence of a frequency dependent out of phase signal (χ”_m_) at low temperatures with a maximum at high frequencies (HF relaxation process, Figure S8). Note that, although in compound **1** the maximum of χ”_m_ is located above 10 kHz, the highest frequency we can achieve, we can clearly see a χ”_m_ signal at high frequencies. When a DC field is applied, we can observe in compounds **1**, **2**, **5**, and **6** the appearance of two maxima: one at low frequencies (LF relaxation process) and one at high frequencies (HF relaxation process, Figure S8). In compounds **3** and **4**, the application of a DC field gives rise to the appearance of a frequency dependent signal with a maximum at around 3 × 10^2^ Hz (Figure S8). In all cases (except for the HF signal in compound **5**), the maxima of the χ”_m_ signals increase in intensity and shift to lower frequencies with increasing the DC field up to a certain DC field. Above this value, the maxima shift to higher frequencies and decrease in intensity (Figure S8). In compound **5**, the HF signal only shows the decrease in intensity and the shift to higher frequencies as the DC field increases.

By fitting the frequency dependence of the χ”_m_ signal to the Debye model with one (in **3** and **4**)^51^ or two (in **1**, **2**, **5**, and **6**)^52^ relaxation processes, we can obtain the relaxation times for the LF (τ_LF_) and HF (τ_HF_) processes at each applied DC field (solid lines in Figure S8). Note that this double relaxation process has been observed in other Dy^III^-containing compounds,^53^ and, since there is only one independent Dy^III^ ion in **1**, **2**, and **5**, it has to be attributed to the presence of two relaxation pathways via excited states.^54^ Only in compound **6** the presence of two relaxation times may be attributed to the presence of two crystallographically independent Dy^III^ centers. Note that the relaxation times obtained at high temperatures in some cases have to be taken with caution since the maxima in the χ”_m_*vs* frequency plot cannot be observed.

As expected, the relaxation times increase as the DC field increases, reach a maximum at intermediate fields, and show a decrease for higher DC fields (Figure S9). In order to perform a detailed study of the χ”_m_ signal as a function of the temperature, we have fixed the DC fields to the values where the relaxation time shows a maximum. Additionally, we have also performed the study of the χ”_m_ signal as a function of the temperature with zero DC field in those compounds that show a χ”_m_ signal with zero DC field (**1**, **2**, and **5**).

**Figure 5:**
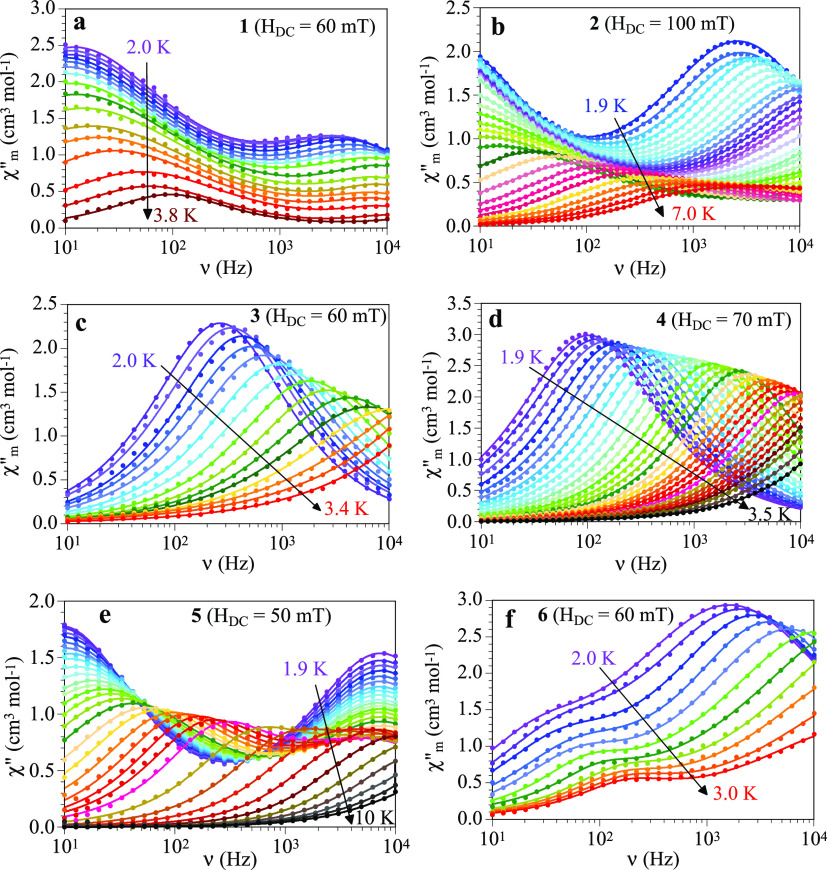
(a–f) Frequency dependence of χ”_m_ at different temperatures for compounds **1**–**6** with different applied DC fields. Solid lines are the best fit to a Debye model with one (in **3** and **4**) or two (in **1**, **2**, **5**, and **6**) relaxation processes.

The frequency dependence of χ”_m_ at different temperatures for compound **1** with zero DC field shows a signal whose maximum appears above 10 kHz (the maximum frequency that we can reach, Figure S10). As the temperature increases, the signal moves to higher frequencies, and, thus, we cannot perform a detailed study of this signal for *H*_DC_ = 0 mT. When a DC field of 60 mT is applied, there are two clear maxima that shift to higher frequencies as the temperature increases (Figure 5a). The χ”_m_ signal can be fitted in the temperature range 2.0–3.8 K with a Debye model considering two relaxation processes: one at low frequencies, LF, and a second one at high frequencies, HF (solid lines in Figure 5a). Finally, we have also performed the AC measurements with an applied DC of 100 mT (Figure S10). These measurements show very clearly how the LF signal shifts to high frequencies as the temperature increases. The corresponding fit to the Debye model for two relaxation processes is shown as solid lines in Figure S10.

Compound **2** shows a similar behavior (Figure 5b): There is only one relaxation process when *H*_DC_ = 0 mT (Figure S11.left), although now the maximum appears below 10 kHz and, therefore, we can fit it to a single relaxation process with the Debye model (solid lines in Figure S11.left). When a DC field of 50 or 100 mT is applied (Figure S11.right and Figure 5b, respectively) we can observe two maxima that can be well reproduced (solid lines in Figure 5b) with a Debye model considering two relaxation processes.

Compounds **3** and **4** show no χ”_m_ signal for *H*_DC_ = 0 mT, but for *H*_DC_ = 60 mT (for **3**) and 70 mT (for **4**) they show a χ”_m_ signal at low temperatures that can be well reproduced with the Debye model for a single relaxation process (solid lines in Figure 5c for **3** and Figure 5d for **4**).

Compound **5** behaves as **2**: it shows one relaxation process for *H*_DC_ = 0 mT (Figure S12) with maxima below 10 kHz that can be fitted to a Debye model with a single relaxation process (solid lines in Figure S12). When a DC field of 50 mT is applied (Figure 5e), we observe two maxima that can be well reproduced (solid lines in Figure 5e) with the Debye model considering two relaxation processes.

Finally, compound **6** shows no χ”_m_ signal for *H*_DC_ = 0 mT, but when a DC field of 60 mT is applied (Figure 5f), we observe two broad maxima that can be well reproduced (solid lines in Figure 5f) with the Debye model considering two relaxation processes.

**Figure 6:**
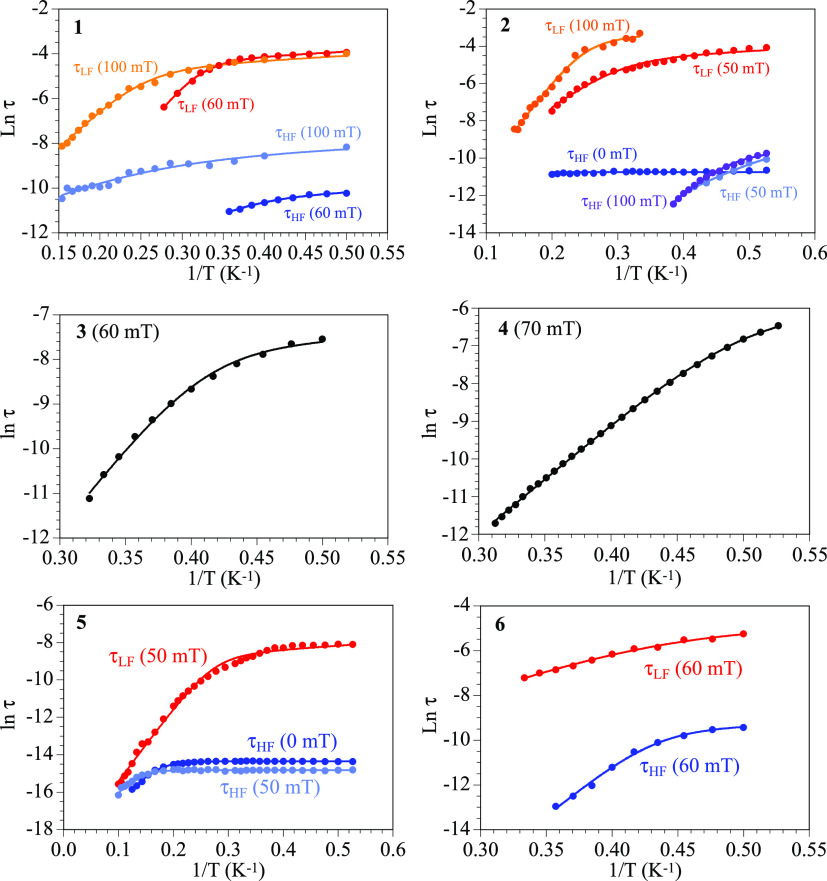
Arrhenius plots of the relaxation times of compounds **1**–**6** with different applied DC fields. Solid lines are the best fits to the general model (eq 1) with the parameters shown in Table 5.

The fit of the χ”_m_ signals shown in Figure 5 (and Figures S10–S12) provides the relaxation times of each process as a function of the temperature. The Arrhenius plots (Ln τ *vs* 1/*T*) of the relaxation times for the different processes with different applied DC fields are shown in Figure 6.

In general, the relaxation times show a straight line behavior at high temperatures (Orbach activated mechanism) with a curvature at lower temperatures (direct mechanism, that appears when *H*_DC_ ≠ 0). Thus, these Arrhenius plots can be fitted to a general model including quantum tunneling (QTM, first term), Orbach (second term), Raman (third term), and direct (fourth term):^55^
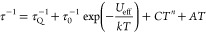
1

In all cases, when *H*_DC_ ≠ 0, the relaxation times can be well fitted with direct and Orbach contributions with the parameters shown in Table 5. In compounds **2** for *H*_DC_ = 0, the relaxation time of the only signal observed can be fitted to a simple quantum tunneling mechanism, whereas in compound **5** the HF signal for *H*_DC_ = 0 and 50 mT can be fitted with a quantum tunneling plus an Orbach mechanism. Note that the temperature ranges are limited by the possibility to obtain reliable fits of the frequency dependence of the χ”_m_ signal.

**Table 5:** Magnetic Parameters from the Fit of the Thermal Dependence of the Relaxation Times in Compounds **1**–**6**

compound	*H*_DC_ (mT)	process	*A* (K^–1^)	τ_0_ (s)	*U*_eff_ (K)
**1**	60	LF	24.8(3)	2.4(1) × 10^–9^	49(1)
	60	HF	1.30(4) × 10^4^	4.28(5) × 10^–9^	25(3)
	100	LF	30(1)	1.2(3) × 10^–6^	36(1)
	100	HF	1.9(2) × 10^3^	4.5(3) × 10^–6^	16(3)
**2**	0	HF	–	2.2(1) × 10^–5^^a^	–
	50	LF	35(1)	3.4(1) × 10^–6^	27(2)
	50	HF	5.6(3) × 10^3^	4.4(7) × 10^–9^	19(4)
	100	LF	10.6(8)	2.6(8) × 10^–7^	46(2)
	100	HF	8.4(5) × 10^3^	1.9(1) × 10^–11^	32(1)
**3**	60	HF	9.7(5) × 10^2^	1.1(6) × 10^–10^	37(1)
**4**	70	HF	2.37(8) × 10^2^	9.0(6) × 10^–10^	29.4(2)
**5**	0	HF	–	5.87(3) × 10^–7^^a^	40.2(8)
			–	1.1(1) × 10^–9^	
	50	LF	1.77(9) × 10^3^	4.1(7) × 10^–9^	39(1)
	50	HF	–	3.65(3) × 10^–7^^a^	48(3)
			–	1.2(3) × 10^–9^	
**6**	60	LF	77(9)	1.0(6) × 10^–6^	20(2)
	60	HF	5.8(5) × 10^3^	1.0(9) × 10^–13^	47(3)

a τ_QT_.

## Conclusions

We have shown the versatility of ethylene glycol as a solvent and as a ligand to prepare coordination polymers with anilato-type ligands and Dy^III^. The possibility of ethylene glycol to act as monodentate (eg-κ*O*), bidentate chelate (eg-κ^2^*O,O’*), and bidentate bridge (μ-eg-1κ*O*,2κ*O’*) has been exploited in this work to prepare up to six different Dy-anilato coordination polymers where the Dy^III^ are surrounded by three chelate anilato ligand that connect each Dy^III^ ion to three neighboring Dy^III^ ions. Additionally, the Dy^III^ ions complete their nona-coordination with chelate, terminal, and/or bridging eg molecules, H_2_O, and/or MeOH.

Thus, with the 2,5-dihydroxy-1,4-benzoquinone dianion (dhbq^2–^), we have prepared a 3D polymer with a diamond-like topology where the Dy^III^ ions complete their coordination with a chelate eg molecule and a bridging eg that forms the fourth Dy–Dy connection in the diamond-like topology (compound **1**). When using the 3,6-dichloro-2,5-dihydroxy-1,4-benzoquinone dianion (chloranilato), we obtain two different coordination polymers depending on the synthetic conditions: a 2D (6,3)-gon brick-wall topology where the Dy^III^ ions complete their coordination with a chelate and a terminal eg molecule (compound **2**) and a 3D diamond-like topology similar to that of compound **1**, although now the chelate eg has been replaced by two water molecules (compound **3**). Finally, when using the 3,6-dibromo-2,5-dihydroxy-1,4-benzoquinone dianion (bromanilato), we obtain up to three different coordination polymers: a 2D (6,3)-gon herringbone topology where the Dy^III^ ions complete their coordination with a chelate eg molecule and a MeOH molecule (compound **4**), a 2D (6,3)-gon brick-wall topology where the Dy^III^ ions complete their coordination with a chelate and a terminal eg molecule (compound **5**), and a 2D (6,3)-gon brick-wall topology with two independent Dy^III^ ions: one with a chelate and a terminal eg molecule and the second one with a chelate eg molecule and a water molecule (compound **6**). The coordination environments of the Dy^III^ ions are tricapped trigonal prisms in compounds **1**–**4** and capped square antiprisms in **5** and **6**.

The competition of the coordination ability of the three coligands used (ethylene glycol, H_2_O and MeOH) results in important changes in the dimensionality and final structures in compounds **1**–**6**.

Thus, if we add water in the synthesis of compound **2**, we observe the formation of compound **3** where each Dy^III^ ion has two coordinated water molecules (instead of a chelate eg, as observed in **2**). This change reduces the steric hindrance around the Dy^III^ centers and allows the formation of an ethylene glycol bridge connecting two Dy^III^ ions that results in a 3D structure in **3** (in contrast to the 2D structure in **2**). In compounds **4**–**6**, prepared with bromanilato, we also observe an important influence of the water molecules. When no water molecule is added, we obtain compound **4**, that contains a coordinated MeOH molecule (besides a chelate eg ligand). When we add water, the MeOH molecule is probably replaced initially by a water molecule leading to a change in the coordination geometry (from tricapped trigonal prismatic to capped square antiprismatic). This change in the geometry reduces the steric hindrance around the Dy^III^ center and allows the inclusion of a terminal eg ligand, as observed in the kinetic phase (compound **5**) and of a water molecule in half of the Dy^III^ centers in the thermodynamic phase (compound **6**).

The magnetic properties of the six coordination polymers are the expected ones for isolated Dy^III^ ions. Interestingly, the six compounds present slow relaxation of the magnetization (in compounds **1**, **2**, and **5** even with no applied DC field). When a DC field is applied, compounds **1**, **2**, **5**, and **6** show two different relaxation processes (high and low frequency processes), whereas compounds **3** and **4** only show one relaxation process. We have studied the frequency dependence of the χ”_m_ signal at different DC fields and determined the optimal DC fields to perform a detailed study of the χ”_m_ signal as a function of the temperature. This study shows that the magnetization of all compounds relaxes through direct and Orbach mechanisms with energy barriers in the range 16–49 K when a DC field is applied and through an Orbach and/or quantum tunneling mechanism when no DC field is applied.

Although there is no a linear correlation, if we compare the *U*_eff_ values for intermediate DC fields (50–70 mT, Table 5) with the distortion from the regular geometry, measured by the shape parameter (Table 4), we can observe an increase in *U*_eff_ as the distortions from the ideal geometry increase. Thus, the more regular compounds (**1** and **2**) show the lower *U*_eff_ values (25 and 19 K, respectively), whereas the most distorted compounds (**3**–**6**) show the highest *U*_eff_ (37, 29.4, 48, and 47 K, respectively). This trend indicates that the higher the distortions of the coordination geometry, the higher is the energy barrier and further confirms that the use of chelating and bulky solvent molecules may be a very adequate strategy to increase the energy barrier in these Dy^III^-based 2D and 3D SIMs.

In summary, we have shown the versatility of the solvent and ligand ethylene glycol to prepare coordination polymers with different dimensionalities (2D and 3D) using Dy^III^ and three different anilato-type ligands (dhbq^2–^, chloranilato, and bromanilato) by slightly changing the synthetic conditions.

Of course, this result opens the way to prepare many other coordination polymers with other interesting magnetic and optical properties by simply changing the Dy^III^ ion by other lanthanoid ions and transition metal ions. We are also extending this study with Dy^III^ (and other ions) using other symmetric and asymmetric anilato-type ligands with X = F, I, CH_3_, Cl/CN, Cl/NO_2_, etc.

## Supporting Information Available

Figures with structural details of compounds **2**–**6**. Tables with bond distances and angles for compounds **1**–**6**. Figures with detailed DC and AC magnetic measurements and a table with the magnetic parameters for compounds **1**–**6** (PDF)

## References

[ref1] Batten S. R.; Champness N. R.; Chen X.-M.; Garcia-Martinez J.; Kitagawa S.; Ohrstrom L.; O’Keeffe M.; Paik Suh M.; Reedijk J. Terminology of metal–organic Frameworks and Coordination Polymers (IUPAC Recommendations 2013). Pure Appl. Chem. 2013, 85, 1715–1724. 10.1351/PAC-REC-12-11-20.

[ref2] Zhang X.; Chen Z.; Liu X.; Hanna S. L.; Wang X.; Taheri-Ledari R.; Maleki A.; Li P.; Farha O. K. A Historical Overview of the Activation and Porosity of metal–organic Frameworks. Chem. Soc. Rev. 2020, 49, 7406–7427. 10.1039/D0CS00997K. 32955065

[ref3] Sessoli R.; Gatteschi D.; Caneschi A.; Novak M. A. Magnetic Bistability in a Metal-Ion Cluster. Nature 1993, 365, 141–143. 10.1038/365141a0.

[ref4] Bagai R.; Christou G. The Drosophila of Single-Molecule Magnetism: [Mn_12_O_12_(O_2_CR)_16_(H_2_O)_4_]. Chem. Soc. Rev. 2009, 38, 1011–1026. 10.1039/b811963e. 19421579

[ref5] Ishikawa N.; Sugita M.; Ishikawa T.; Koshihara S. Y.; Kaizu Y. Lanthanide Double-Decker Complexes Functioning as Magnets at the Single-Molecular Level. J. Am. Chem. Soc. 2003, 125, 8694–8695. 10.1021/ja029629n. 12862442

[ref6] Sessoli R.; Powell A. K. Strategies Towards Single Molecule Magnets Based on Lanthanide Ions. Coord. Chem. Rev. 2009, 253, 2328–2341. 10.1016/j.ccr.2008.12.014.

[ref7] Dey A.; Kalita P.; Chandrasekhar V. Lanthanide(III)-Based Single-Ion Magnets. ACS Omega 2018, 3, 9462–9475. 10.1021/acsomega.8b01204. 31459081 PMC6644820

[ref8] Fordham S.; Wang X.; Bosch M.; Zhou H. Lanthanide Metal-Organic Frameworks: Syntheses, Properties, and Potential Applications. Struct. Bonding (Berlin) 2014, 163, 1–27. 10.1007/430_2014_162.

[ref9] Wang C.; Liu X.; Keser Demir N.; Chen J. P.; Li K. Applications of Water Stable Metal-Organic Frameworks. Chem. Soc. Rev. 2016, 45, 5107–5134. 10.1039/C6CS00362A. 27406473

[ref10] Liu X.; Fu W.; Bouwman E. One-Step Growth of Lanthanoid Metal-Organic Framework (MOF) Films Under Solvothermal Conditions for Temperature Sensing. Chem. Commun. 2016, 52, 6926–6929. 10.1039/C6CC01407K. 27147478

[ref11] Zeng M. H.; Yin Z.; Tan Y. X.; Zhang W. X.; He Y. P.; Kurmoo M. Nanoporous Cobalt(II) MOF Exhibiting Four Magnetic Ground States and Changes in Gas Sorption upon Post-Synthetic Modification. J. Am. Chem. Soc. 2014, 136, 4680–4688. 10.1021/ja500191r. 24588716

[ref12] Kurmoo M. Magnetic Metal-Organic Frameworks. Chem. Soc. Rev. 2009, 38, 1353–1379. 10.1039/b804757j. 19384442

[ref13] Coronado E.; Minguez Espallargas G. Dynamic Magnetic MOFs. Chem. Soc. Rev. 2013, 42, 1525–1539. 10.1039/C2CS35278H. 23146915

[ref14] Campo J.; Falvello L. R.; Forcen-Vazquez E.; Saenz de Pipaon C.; Palacio F.; Tomas M. A Symmetric, Triply Interlaced 3-D Anionic MOF that Exhibits both Magnetic Order and SMM Behaviour. Dalton Trans. 2016, 45, 16764–16768. 10.1039/C6DT02652D. 27602786

[ref15] Brunet G.; Safin D. A.; Jover J.; Ruiz E.; Murugesu M. Single-Molecule Magnetism Arising from Cobalt(II) Nodes of a Crystalline Sponge. J. Mater. Chem. C 2017, 5, 835–841. 10.1039/C6TC04703C.

[ref16] Rinehart J. D.; Long J. R. Exploiting Single-Ion Anisotropy in the Design of f-Element Single-Molecule Magnets. Chem. Sci. 2011, 2, 2078–2085. 10.1039/c1sc00513h.

[ref17] Kitagawa S.; Kawata S. Coordination Compounds of 1,4-Dihydroxybenzoquinone and its Homologues. Structures and Properties. Coord. Chem. Rev. 2002, 224, 11–34. 10.1016/S0010-8545(01)00369-1.

[ref18] Mercuri M. L.; Congiu F.; Concas G.; Sahadevan S. A. Recent Advances on Anilato-Based Molecular Materials with Magnetic and/or Conducting Properties. Magnetochemistry 2017, 3, 17 10.3390/magnetochemistry3020017.

[ref19] Atzori M.; Benmansour S.; Mínguez Espallargas G.; Clemente-León M.; Abhervé A.; Gómez-Claramunt P.; Coronado E.; Artizzu F.; Sessini E.; Deplano P.; Serpe A.; Mercuri M. L.; Gómez García C. J. A Family of Layered Chiral Porous Magnets Exhibiting Tunable Ordering Temperatures. Inorg. Chem. 2013, 52, 10031–10040. 10.1021/ic4013284. 23968133

[ref20] Benmansour S.; Abhervé A.; Gómez-Claramunt P.; Vallés-García C.; Gómez-García C. J. Nanosheets of Two-Dimensional Magnetic and Conducting Fe(II)/Fe(III) Mixed-Valence Metal–Organic Frameworks. ACS Appl. Mater. Interfaces 2017, 9, 26210–26218. 10.1021/acsami.7b08322. 28715894

[ref21] Benmansour S.; Gómez-García C. J. Heterometallic Anilato-Based Layered Magnets. Gen. Chem. 2020, 6, 190033 10.21127/yaoyigc20190033.

[ref22] Riley P. E.; Haddad S. F.; Raymond K. N. Preparation of Praseodymium(III) Chloranilate and the Crystal Structures of Pr_2_(C_6_Cl_2_O_4_)_3_.8C_2_H_5_OH and Na_3_[C_6_H_2_O(OH)(SO_3_)_2_].H_2_O. Inorg. Chem. 1983, 22, 3090–3096. 10.1021/ic00163a022.

[ref23] Robl C. Complexes with Substituted 2,5-Dihydroxy-p-Benzoquinones: The Inclusion Compounds [Y(H_2_O)_3_]_2_(C_6_Cl_2_O_4_)_3_]·6.6H_2_O and [Y(H_2_O)_3_]_2_(C_6_Br_2_O_4_)_3_]·6H_2_O. Mater. Res. Bull. 1987, 22, 1483–1491. 10.1016/0025-5408(87)90213-3.

[ref24] Abrahams B. F.; Coleiro J.; Ha K.; Hoskins B. F.; Orchard S. D.; Robson R. Dihydroxybenzoquinone and Chloranilic Acid Derivatives of Rare Earth Metals. J. Chem. Soc., Dalton Trans. 2002, 1586–1594. 10.1039/b109296k.

[ref25] Benmansour S.; López-Martínez G.; Canet-Ferrer J.; Gómez-García C. J. A Family of Lanthanoid Dimers with Nitroanilato Bridges. Magnetochemistry 2016, 2, 32 10.3390/magnetochemistry2030032.

[ref26] Benmansour S.; Pérez-Herráez I.; López-Martínez G.; Gómez García C. J. Solvent-Modulated Structures in Anilato-Based 2D Coordination Polymers. Polyhedron 2017, 135, 17–25. 10.1016/j.poly.2017.06.052.

[ref27] Gómez-Claramunt P.; Benmansour S.; Hernández-Paredes A.; Cerezo-Navarrete C.; Rodríguez-Fernández C.; Canet-Ferrer J.; Cantarero A.; Gómez-García C. J. Tuning the Structure and Properties of Lanthanoid Coordination Polymers with an Asymmetric Anilato Ligand. Magnetochemistry 2018, 4, 6 10.3390/magnetochemistry4010006.

[ref28] Benmansour S.; Hernández-Paredes A.; Gómez-García C. J. Effect of the Lanthanoid-Size on the Structure of a Series of Lanthanoid-Anilato 2-D Lattices. J. Coord. Chem. 2018, 71, 845–863. 10.1080/00958972.2017.1420182.

[ref29] Benmansour S.; Pérez-Herráez I.; Cerezo-Navarrete C.; López-Martínez G.; Martínez Hernandez C.; Gómez-García C. J. Solvent-Modulation of the Structure and Dimensionality in Lanthanoid-Anilato Coordination Polymers. Dalton Trans 2018, 47, 6729–6741. 10.1039/C8DT00143J. 29713717

[ref30] Benmansour S.; Hernández-Paredes A.; Gómez-García C. J. Two Dimensional Magnetic Coordination Polymers Formed by Lanthanoids and Chlorocyananilato. Magnetochemistry 2018, 4, 58 10.3390/magnetochemistry4040058.

[ref31] Hernández-Paredes A.; Cerezo-Navarrete C.; Gómez García C. J.; Benmansour S. Slow Relaxation in Doped Coordination Polymers and Dimers Based on Lanthanoids and Anilato Ligands. Polyhedron 2019, 170, 476–485. 10.1016/j.poly.2019.06.004.

[ref32] Benmansour S.; Hernández-Paredes A.; Mondal A.; López Martínez G.; Canet-Ferrer J.; Konar S.; Gómez-García C. J. Slow Relaxation of the Magnetization, Reversible Solvent Exchange and Luminescence in 2D Anilato-Based Frameworks. Chem. Commun. 2020, 56, 9862–9865. 10.1039/D0CC03964K. 32840511

[ref33] Benmansour S.; Gómez-García C. J. Lanthanoid-Anilato Complexes and Lattices. Magnetochemistry 2020, 6, 71 10.3390/magnetochemistry6040071.

[ref34] Benmansour S.; Hernández-Paredes A.; Bayona-Andrés M.; Gómez-García C. J. Slow Relaxation of the Magnetization in Anilato-Based Dy(III) 2D Lattices Molecules 2021, 26.1190 10.3390/molecules26041190 33672166 PMC7926458

[ref35] Benmansour S.; Gómez-García C. J.; Hernández-Paredes A. The Complete Series of Lanthanoid-Chloranilato Lattices with Dimethylsulfoxide: Role of the Lanthanoid Size on the Coordination Number and Crystal Structure. Crystals 2022, 12, 261 10.3390/cryst12020261.

[ref36] Sahadevan S. A.; Monni N.; Abhervé A.; Cosquer G.; Oggianu M.; Ennas G.; Yamashita M.; Avarvari N.; Mercuri M. L. Dysprosium Chlorocyanoanilate-Based 2D-Layered Coordination Polymers. Inorg. Chem. 2019, 58, 13988–13998. 10.1021/acs.inorgchem.9b01968. 31566958

[ref37] Bondaruk K.; Hua C. Effect of Counterions on the Formation and Structures of Ce(III) and Er(III) Chloranilate Frameworks. Cryst. Growth Des 2019, 19, 3338–3347. 10.1021/acs.cgd.9b00233.

[ref38] Diaz-Torres R.; Alvarez S. Coordinating Ability of Anions and Solvents Towards Transition Metals and Lanthanides. Dalton Trans 2011, 40, 10742–10750. 10.1039/c1dt11000d. 21927754

[ref39] Alvarez S. Coordinating Ability of Anions, Solvents, Amino Acids, and Gases Towards Alkaline and Alkaline-Earth Elements, Transition Metals, and Lanthanides. Chem.—Eur. J. 2020, 26, 8663–8663. 10.1002/chem.202001687. 32662108

[ref40] Kharitonov A. D.; Trofimova O. Y.; Meshcheryakova I. N.; Fukin G. K.; Khrizanforov M. N.; Budnikova Y. H.; Bogomyakov A. S.; Aysin R. R.; Kovalenko K. A.; Piskunov A. V. 2D-metal–organic Coordination Polymers of Lanthanides (La(III), Pr(III) and Nd(III)) with Redox-Active Dioxolene Bridging Ligands. CrystEngComm 2020, 22, 4675–4679. 10.1039/D0CE00767F.

[ref41] Artizzu F.; Atzori M.; Liu J.; Mara D.; Van Hecke K.; Van Deun R. Solution-Processable Yb/Er 2D-Layered Metallorganic Frameworks with High NIR-Emission Quantum Yields. J. Mater. Chem. C 2019, 7, 11207–11214. 10.1039/C9TC03698A.

[ref42] Kingsbury C. J.; Abrahams B. F.; Auckett J. E.; Chevreau H.; Dharma A. D.; Duyker S.; He Q.; Hua C.; Hudson T. A.; Murray K. S.; Phonsri W.; Peterson V. K.; Robson R.; White K. F. Square Grid Metal-Chloranilate Networks as Robust Host Systems for Guest Sorption. Chem.—Eur. J. 2019, 25, 5222–5234. 10.1002/chem.201805600. 30729591

[ref43] Hua C.; Tay H. M.; He Q.; Harris T. D. A Series of Early Lanthanide Chloranilate Frameworks with a Square Grid Topology. Aust. J. Chem. 2019, 72, 778–785. 10.1071/CH19193.

[ref44] Bain G. A.; Berry J. F. Diamagnetic Corrections and Pascal’s Constants. J. Chem. Educ. 2008, 85, 532–536. 10.1021/ed085p532.

[ref45] Crysalispro, 171.33.55; Oxford Diffraction, 2004.

[ref46] Sheldrick G. M. Crystal Structure Refinement with SHELXL. Acta Cryst. C 2015, 71, 3–8. 10.1107/S2053229614024218. PMC4294323 25567568

[ref47] Dolomanov O. V.; Bourhis L. J.; Gildea R. J.; Howard J. A. K.; Puschmann H. OLEX2: A Complete Structure Solution, Refinement and Analysis Program. J. Appl. Crystallogr. 2009, 42, 339–341. 10.1107/S0021889808042726.

[ref48] Sheldrick G. M. A Short History of SHELX. Acta Cryst. A 2008, 64, 112–122. 10.1107/S0108767307043930. 18156677

[ref49] Álvarez S. Distortion Pathways of Transition Metal Coordination Polyhedra Induced by Chelating Topology. Chem. Rev. 2015, 115, 13447–13483. 10.1021/acs.chemrev.5b00537. 26575868

[ref50] Sorace L.; Gatteschi D. In Electronic Structure and Magnetic Properties of Lanthanide Molecular Complexes; Layfield R. A., Murugesu M., Eds.; Wiley, 2015; Vol. 1, pp 1–25.

[ref51] Aubin S. M. J.; Sun Z.; Pardi L.; Krzystek J.; Folting K.; Brunel L.; Rheingold A. L.; Christou G.; Hendrickson D. N. Reduced Anionic Mn12 Molecules with Half-Integer Ground States as Single-Molecule Magnets. Inorg. Chem. 1999, 38, 5329–5340. 10.1021/ic990613g.

[ref52] Dolai M.; Ali M.; Titis J.; Boca R. Cu(II)-Dy(III) and Co(III)-Dy(III) Based Single Molecule Magnets with Multiple Slow Magnetic Relaxation Processes in the Cu(II)-Dy(III) Complex. Dalton Trans 2015, 44, 13242–13249. 10.1039/C5DT00960J. 26121421

[ref53] Ruiz J.; Mota A. J.; Rodriguez-Dieguez A.; Titos S.; Herrera J. M.; Ruiz E.; Cremades E.; Costes J. P.; Colacio E. Field and Dilution Effects on the Slow Relaxation of a Luminescent DyO_9_ Low-Symmetry Single-Ion Magnet. Chem. Commun. 2012, 48, 7916–7918. 10.1039/c2cc32518g. 22692369

[ref54] Blagg R. J.; Ungur L.; Tuna F.; Speak J.; Comar P.; Collison D.; Wernsdorfer W.; McInnes E. J. L.; Chibotaru L. F.; Winpenny R. E. P. Magnetic Relaxation Pathways in Lanthanide Single-Molecule Magnets. Nat. Chem. 2013, 5, 673–678. 10.1038/nchem.1707. 23881498

[ref55] Demir S.; Zadrozny J. M.; Long J. R. Large Spin-Relaxation Barriers for the Low-Symmetry Organolanthanide Complexes [Cp*_2_Ln(BPh_4_)] (Cp* = pentamethylcyclo-pentadienyl; Ln = Tb, Dy). Chem.—Eur. J. 2014, 20, 9524–9529. 10.1002/chem.201403751. 24975126

