# Supplementary material for: The Versatility of Ethylene Glycol to Tune the Dimensionality and Magnetic Properties in DyIII-Anilato-Based Single-Ion Magnets
Source: Cryst Growth Des. 2023 Jan 17;23(2):1269–80. doi: 10.1021/acs.cgd.2c01409 (PMC10389752; doi:10.1021/acs.cgd.2c01409)
Supplement: Supplementary file 1 — cg2c01409_si_001.pdf [file cg2c01409_si_001.pdf]

Supporting Information

The versatility of ethylene glycol to tune the dimensionality and magnetic properties in Dy(III)-anilato-based single-ion magnets

Samia Benmansour*, Cristina Pintado-Zaldo, Javier Martínez-Ponce, Antonio Hernández-Paredes, Antonio Valero-Martínez, Miriam Gómez-Benmansour, Carlos J Gómez-García*.

Departamento de Química Inorgánica. Universidad de Valencia. Dr. Moliner 50, 46100. Burjasot (Valencia) Spain. E-mail: sam.ben@uv.es (S.B.); carlos.gomez@uv.es (C.J.G.-G.)

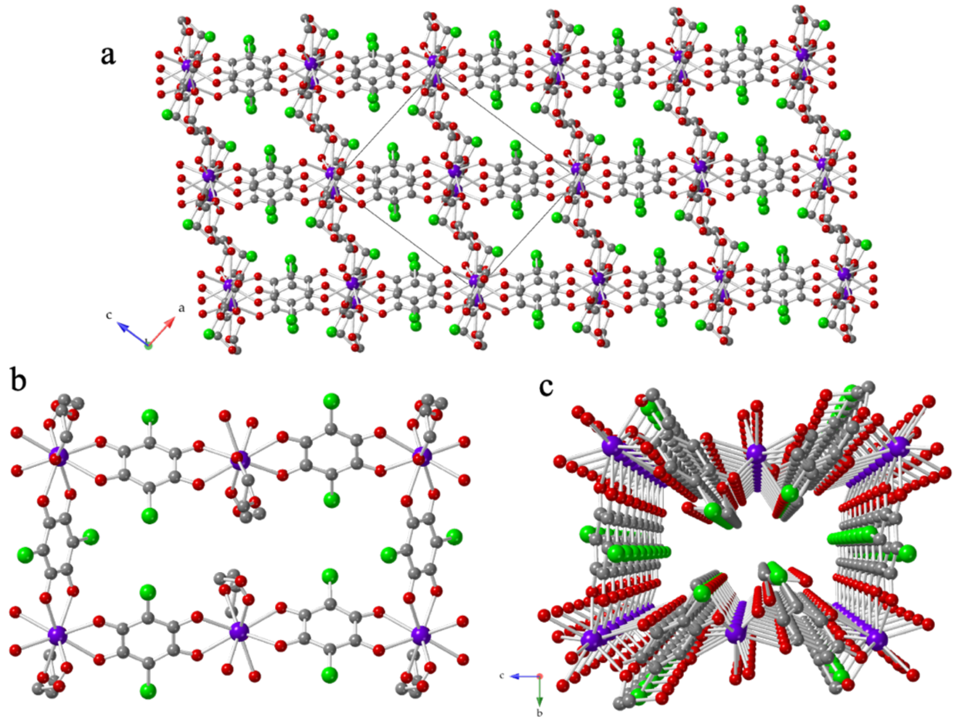

Figure S1. *Structure of compound **2**. (**a**) View of three consecutive layers parallel to the 101 plane. (**b**) View of a six-membered rectangular cavity. (**c**) Perspective view of a rectangular channel along the a direction. Color code: Dy = purple, C = grey, O = red and Cl = green.*

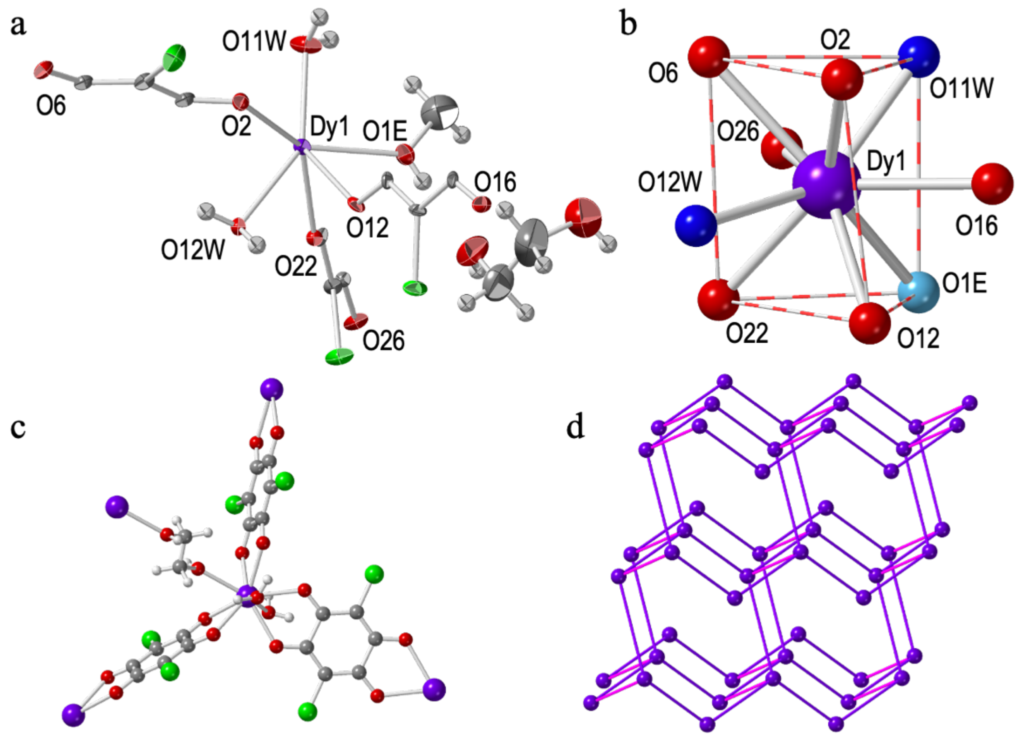

Figure S2. *Structure of compound* ***3***: ***(a)*** *Asymmetric unit with labelling scheme. Ellipsoids are drawn at 50 % probability.* ***(b)*** *Tricapped trigonal prismatic coordination geometry of the Dy(III) ion. O atoms from water and ethylene glycol are displayed in dark and light blue, respectively.* ***(c)*** *Complete coordination environment around the Dy(III) ions.* ***(d)*** *3D diamond-type structure. Purple and pink lines represent anilato and ethylene glycol bridges, respectively. Color code: Dy = purple, C = grey, O = red and Cl = green.*

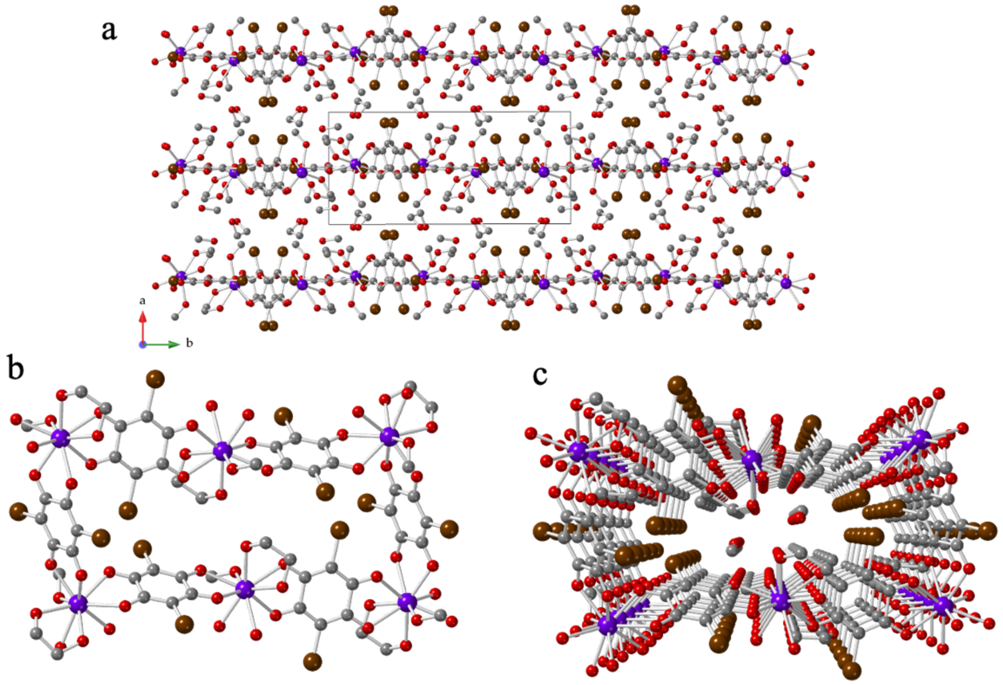

Figure S3. *Structure of compound* ***4***. (***a***) *View of three consecutive layers parallel to the bc plane.* (***b***) *View of a six-membered rectangular cavity.* (***c***) *Perspective view of a rectangular channel along the a direction. Color code: Dy = purple, C = grey, O = red and Br = brown.*

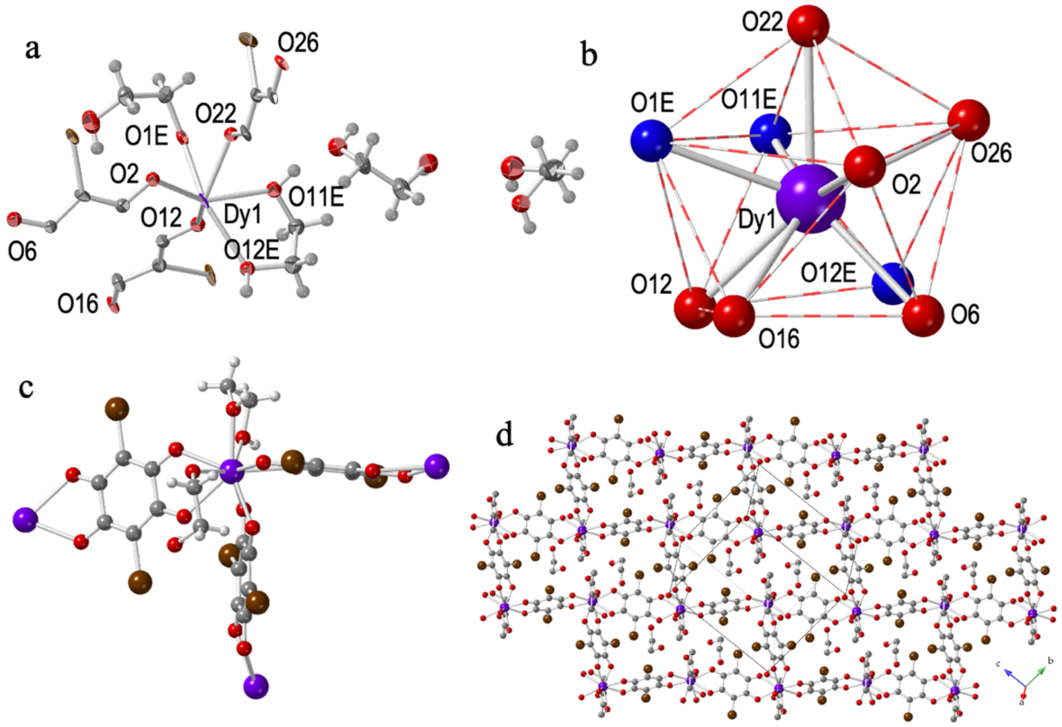

Figure S4. *Structure of compound* ***5***: ***(a)*** *Asymmetric unit with labelling scheme. Ellipsoids are drawn at 60 % probability.* ***(b)*** *Caped square antiprismatic coordination geometry of the Dy(III) ion. O atoms from ethylene glycol are displayed in dark blue.* ***(b)*** *Complete coordination environment around the Dy(III) ions.* ***(d)*** *View of one layer showing the 2D brick-wall type structure. Color code: Dy = purple, C = grey, O = red and Br = brown.*

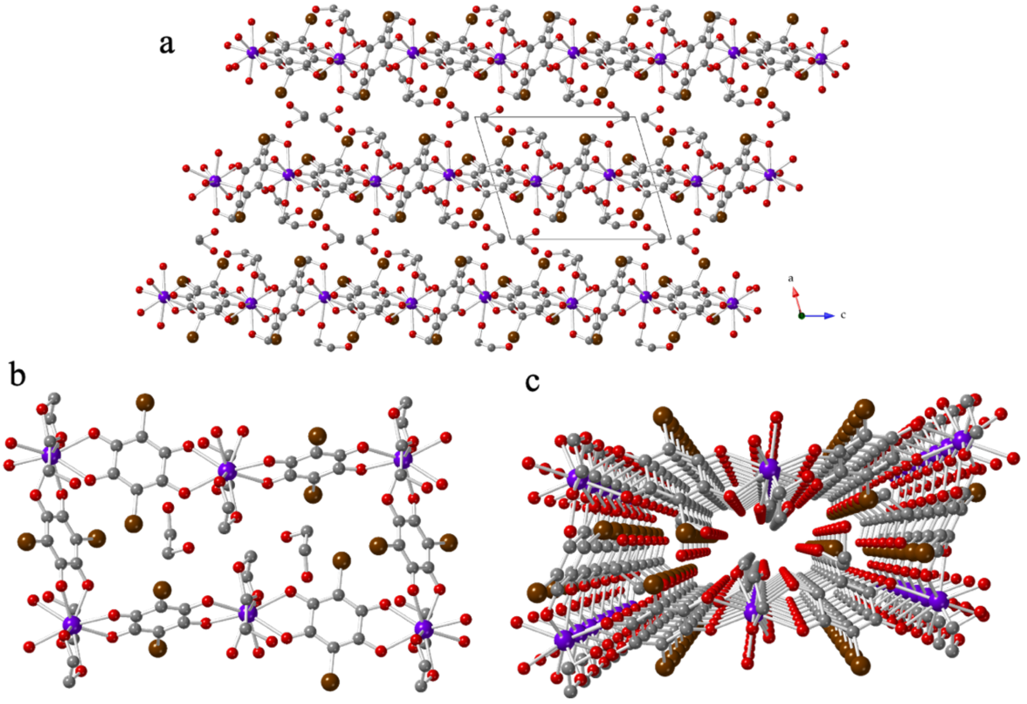

Figure S5. *Structure of compound* ***5***. *(**a**) View of three consecutive layers parallel to the bc plane.* ***(b)*** *View of a six-membered rectangular cavity.* ***(c)*** *Perspective view of a rectangular channel along the a direction. Color code: Dy = purple, C = grey, O = red and Br = brown.*

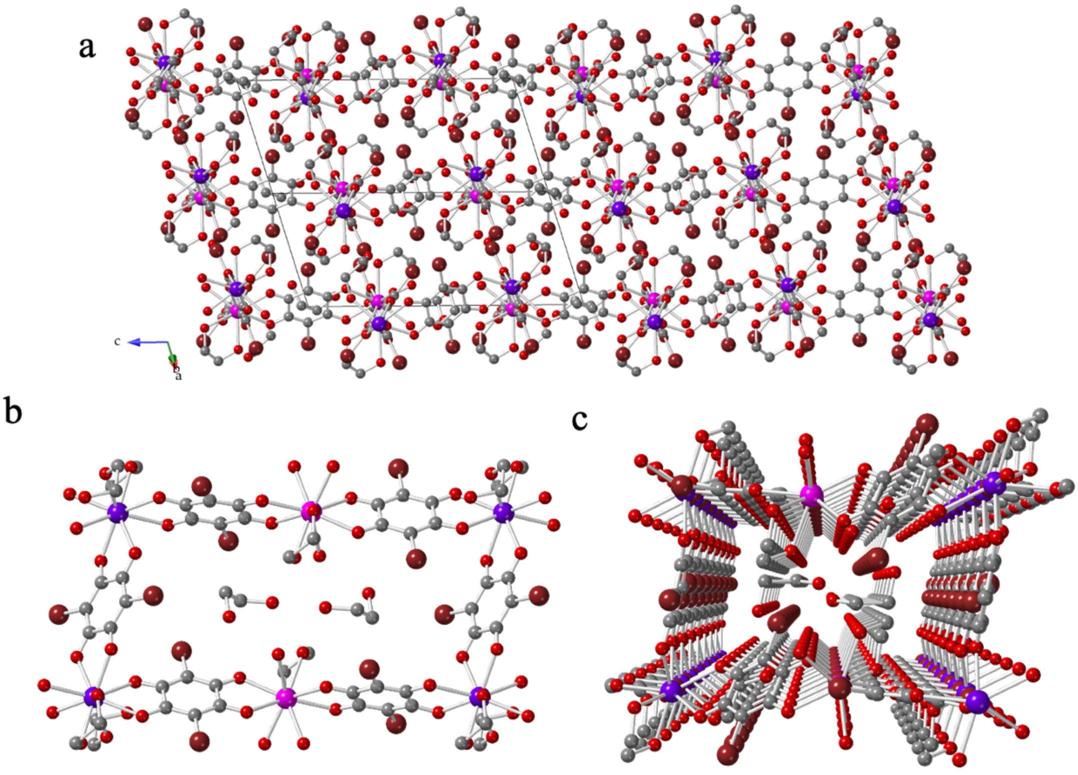

Figure S6. *Structure of compound **6**. (**a**) View of three consecutive layers parallel to the (101) plane. (**b**) View of a six-membered rectangular cavity. (**c**) Perspective view of a rectangular channel along the a direction. Color code: Dy = purple, C = grey, O = red and Br = brown.*

Table S1. Bond distances for [Dy_2(dhbq)$_3$(eg-k^2*O,O'*)$_2$(μ-eg-k*O*)]·4eg·$2H_2O$ (**1**).

Atom	Atom	Distance (Å)	Atom	Atom	Distance (Å)	Atom	Atom	Distance (Å)
Dy1	O22	2.399(8)	O2E	C2E	1.431(17)	C1	C2	1.392(15)
Dy1	O2	2.330(9)	O26	C23	1.267(14)	C1	C6	1.399(18)
Dy1	O16^1	2.393(9)	O6	C6	1.243(15)	C2	C6^3	1.543(17)
Dy1	O2E	2.421(9)	O12	C12	1.271(14)	O201	C201	1.42(2)
Dy1	O26^2	2.373(8)	O11E	C11E	1.453(16)	C2E	C1E	1.490(19)
Dy1	O6^3	2.441(9)	O1E	C1E	1.424(18)	O202	C202	1.40(2)
Dy1	O12	2.384(9)	C21	C22	1.393(14)	C12	C11	1.414(17)
Dy1	O11E	2.466(9)	C21	C23	1.368(16)	O102	C102	1.44(2)
Dy1	O1E	2.452(10)	C22	C23^2	1.542(16)	C101	C102	1.43(2)
O22	C22	1.262(14)	C16	C12^1	1.516(17)	C202	C201	1.59(3)
O2	C2	1.265(16)	C16	C11	1.378(17)	C11E	C11E^4	1.49(3)
O16	C16	1.269(14)	O101	C101	1.39(2)			

1 = -x, 2-y, 1-z; 2 = -x, 1-y, -z; 3 = 1-x, 1-y, 1-z; 4 = -x, 1-y, 1 – z.

Table S2. Bond Angles for [Dy_2(dhbq)$_3$(eg-k^2*O,O'*)$_2$(μ-eg-k*O*)]·4eg·$2H_2O$ (**1**).

Atom	Atom	Atom	Angle (˚)	Atom	Atom	Atom	Angle (º)	Atom	Atom	Atom	Angle (º)
O22	Dy1	O2E	72.5(3)	O26^3	Dy1	O11E	95.0(3)	O26	C23	C21	125.1(11)
O22	Dy1	O6^1	71.8(3)	O26^3	Dy1	O1E	78.0(3)	O26	C23	C22^3	114.7(10)
O22	Dy1	O11E	140.4(3)	O6^1	Dy1	O11E	68.9(3)	C21	C23	C22^3	120.1(10)
O22	Dy1	O1E	73.1(3)	O6^1	Dy1	O1E	139.9(3)	O16	C16	C12^2	115.7(10)
O2	Dy1	O22	93.4(3)	O12	Dy1	O22	126.9(3)	O16	C16	C11	124.3(11)
O2	Dy1	O16^2	83.8(3)	O12	Dy1	O16^2	66.5(3)	C11	C16	C12^2	120.0(11)
O2	Dy1	O2E	69.4(3)	O12	Dy1	O2E	121.7(3)	C2	C1	C6	122.2(11)
O2	Dy1	O26^3	136.2(3)	O12	Dy1	O6^1	118.8(3)	O2	C2	C1	126.2(11)
O2	Dy1	O6^1	65.8(3)	O12	Dy1	O11E	70.8(3)	O2	C2	C6^1	114.6(11)
O2	Dy1	O12	139.5(3)	O12	Dy1	O1E	69.9(3)	C1	C2	C6^1	119.2(12)
O2	Dy1	O11E	75.3(3)	O1E	Dy1	O11E	139.9(3)	O2E	C2E	C1E	109.2(12)
O2	Dy1	O1E	135.4(3)	C22	O22	Dy1	119.8(7)	O6	C6	C1	126.4(12)
O16^2	Dy1	O22	143.3(3)	C2	O2	Dy1	123.4(7)	O6	C6	C2^1	115.0(12)
O16^2	Dy1	O2E	72.4(3)	C16	O16	Dy1^2	120.9(8)	C1	C6	C2^1	118.6(11)
O16^2	Dy1	O6^1	136.7(3)	C2E	O2E	Dy1	117.1(7)	O12	C12	C16^2	115.7(10)
O16^2	Dy1	O11E	74.2(3)	C23	O26	Dy1^3	121.3(7)	O12	C12	C11	123.7(11)
O16^2	Dy1	O1E	83.4(3)	C6	O6	Dy1^1	120.0(8)	C11	C12	C16^2	120.6(11)
O2E	Dy1	O6^1	119.5(3)	C12	O12	Dy1	121.2(8)	O1E	C1E	C2E	106.7(12)
O2E	Dy1	O11E	133.2(3)	C11E	O11E	Dy1	116.4(8)	O101	C101	C102	118.9(16)
O2E	Dy1	O1E	65.9(3)	C1E	O1E	Dy1	118.4(8)	C101	C102	O102	109.4(16)
O26^3	Dy1	O22	67.0(3)	C23	C21	C22	121.2(11)	O202	C202	C201	107.6(14)
O26^3	Dy1	O16^2	135.3(3)	O22	C22	C21	125.0(11)	O201	C201	C202	113.1(15)
O26^3	Dy1	O2E	131.8(3)	O22	C22	C23^3	116.4(10)	O11E	C11E	C11E^4	108.7(14)
O26^3	Dy1	O6^1	70.8(3)	C21	C22	C23^3	118.6(10)	C16	C11	C12	119.4(12)
O26^3	Dy1	O12	69.1(3)								

1 = 1-x, 1-y, 1-z; 2 = -x, 2-y, 1-z; 3 = -x, 1-y, -z; 4 = -x, 1-y, 1-z.

Table S3. Bond distances for $[Dy_2(C_6O_4Cl_2)_3(eg\text{-}k^2O,O')_2(eg\text{-}kO)_2]\cdot 2eg\cdot H_2O$ (**2**)

Atom	Atom	Distance (Å)	Atom	Atom	Distance (Å)	Atom	Atom	Distance (Å)
Dy1	O12	2.394(3)	Cl1	C1	1.741(5)	C12	C11	1.401(6)
Dy1	O3	2.361(3)	O12	C12	1.248(5)	C12	C16^{2}	1.536(6)
Dy1	O5^{1}	2.434(3)	O3	C3	1.259(5)	C4	C5	1.396(6)
Dy1	O16^{2}	2.398(3)	O5	C5	1.251(5)	C4	C3	1.411(6)
Dy1	O2	2.467(3)	O16	C16	1.260(5)	C11	C16	1.391(6)
Dy1	O1E	2.409(3)	O2	C2	1.260(5)	C5	C6	1.531(6)
Dy1	O12E	2.409(3)	O1E	C1E	1.427(5)	C6	C1	1.399(6)
Dy1	O6^{1}	2.393(3)	O12E	C12E	1.460(5)	C2	C3	1.530(6)
Dy1	O11E	2.465(3)	O6	C6	1.255(5)	C2	C1	1.389(6)
Cl11	C11	1.740(4)	O11E	C11E	1.446(5)	C12E	C11E	1.489(6)
Cl4	C4	1.730(4)	O2E	C2E	1.432(5)	C1E	C2E	1.494(6)

1 = ½ + x, ½-y, -1/2 + z; 2 = 1-x, 1-y, 1 – z.

Table S4. Bond angles for $[Dy_2(C_6O_4Cl_2)_3(eg\text{-}k^2O,O')_2(eg\text{-}kO)_2]\cdot 2eg\cdot H_2O$ (**2**)

Atom	Atom	Atom	Angle (˚)	Atom	Atom	Atom	Angle (˚)	Atom	Atom	Atom	Angle (˚)
O12	Dy1	O5^{1}	71.04(9)	O12E	Dy1	O1E	82.70(10)	C12	C11	Cl11	118.6(3)
O12	Dy1	O16^{2}	67.15(10)	O12E	Dy1	O11E	65.86(10)	C16	C11	Cl11	119.0(3)
O12	Dy1	O2	71.09(9)	O6^{1}	Dy1	O12	135.94(9)	C16	C11	C12	122.4(4)
O12	Dy1	O1E	93.67(10)	O6^{1}	Dy1	O5^{1}	65.01(9)	O5	C5	C4	125.4(4)
O12	Dy1	O12E	147.96(10)	O6^{1}	Dy1	O16^{2}	94.73(10)	O5	C5	C6	115.4(4)
O12	Dy1	O11E	126.15(10)	O6^{1}	Dy1	O2	144.81(10)	C4	C5	C6	119.1(4)
O3	Dy1	O12	76.11(10)	O6^{1}	Dy1	O1E	74.44(10)	O16	C16	C12^{2}	116.2(4)
O3	Dy1	O5^{1}	124.72(10)	O6^{1}	Dy1	O12E	73.79(10)	O16	C16	C11	125.4(4)
O3	Dy1	O16^{2}	132.27(10)	O6^{1}	Dy1	O11E	73.43(10)	C11	C16	C12^{2}	118.4(4)
O3	Dy1	O2	65.37(10)	O11E	Dy1	O2	71.55(10)	O6	C6	C5	114.7(4)
O3	Dy1	O1E	69.35(10)	C12	O12	Dy1	120.1(3)	O6	C6	C1	125.8(4)
O3	Dy1	O12E	72.83(10)	C3	O3	Dy1	123.7(3)	C1	C6	C5	119.5(4)
O3	Dy1	O6^{1}	133.00(10)	C5	O5	Dy1^{3}	120.5(3)	O2	C2	C3	115.1(4)
O3	Dy1	O11E	119.58(10)	C16	O16	Dy1^{2}	119.7(3)	O2	C2	C1	126.0(4)
O5^{1}	Dy1	O2	135.81(10)	C2	O2	Dy1	120.0(3)	C1	C2	C3	118.9(4)
O5^{1}	Dy1	O11E	115.69(10)	C1E	O1E	Dy1	130.5(2)	O3	C3	C4	124.9(4)
O16^{2}	Dy1	O5^{1}	70.54(9)	C12E	O12E	Dy1	115.7(3)	O3	C3	C2	115.6(4)
O16^{2}	Dy1	O2	74.38(9)	C6	O6	Dy1^{3}	122.6(3)	C4	C3	C2	119.6(4)
O16^{2}	Dy1	O1E	139.78(9)	C11E	O11E	Dy1	119.8(3)	O12E	C12E	C11E	108.9(4)
O16^{2}	Dy1	O12E	132.20(10)	O12	C12	C11	124.3(4)	C6	C1	Cl1	118.2(3)
O16^{2}	Dy1	O11E	66.39(10)	O12	C12	C16^{2}	116.6(4)	C2	C1	Cl1	120.4(4)
O1E	Dy1	O5^{1}	69.80(10)	C11	C12	C16^{2}	119.2(4)	C2	C1	C6	120.9(4)
O1E	Dy1	O2	134.44(10)	C5	C4	Cl4	119.4(3)	O1E	C1E	C2E	112.4(4)
O1E	Dy1	O11E	139.97(10)	C5	C4	C3	120.7(4)	O2E	C2E	C1E	109.7(4)
O12E	Dy1	O5^{1}	134.97(10)	C3	C4	Cl4	119.9(3)	O11E	C11E	C12E	105.8(4)
O12E	Dy1	O2	88.81(10)								

1 = ½ + x, ½-y, -1/2 + z; 2 = 1-x, 1-y, 1-z; 3 = -1/2 + x, ½-y, ½ + z.

Table S5. Bond distances for $[Dy_2(C_6O_4Cl_2)_3(\mu\text{-eg-k}O)(H_2O)_4]\cdot2eg\cdot7H_2O$ (**3**).

Atom	Atom	Distance (Å)	Atom	Atom	Distance (Å)	Atom	Atom	Distance (Å)
Dy1	O12W	2.456(4)	Cl1	C1	1.731(7)	C22	C21	1.397(8)
Dy1	O22	2.413(4)	O22	C22	1.252(8)	C26	C21	1.396(9)
Dy1	O12	2.411(5)	O12	C12	1.268(7)	C11	C16	1.414(9)
Dy1	$O6^1$	2.407(4)	O6	C6	1.269(7)	C11	C12	1.374(9)
Dy1	$O16^2$	2.451(4)	O16	C16	1.256(8)	C6	C1	1.368(9)
Dy1	O2	2.369(4)	O2	C2	1.245(7)	C16	$C12^2$	1.521(8)
Dy1	$O26^3$	2.428(4)	O26	C26	1.258(7)	O101	C101	1.411(15)
Dy1	O1E	2.414(5)	O1E	C1E	1.523(12)	O102	C102	1.426(11)
Dy1	O11W	2.371(4)	C2	$C6^1$	1.548(9)	C102	C101	1.486(17)
Cl21	C21	1.725(6)	C2	C1	1.406(9)	C1E	$C1E^4$	1.35(2)
Cl11	C11	1.741(6)	C22	$C26^3$	1.538(8)			

1 = 2-x, 1-y, -z; 2 = 1-x, 1-y, 1-z; 3 = 2-x, 2-y, 1-z; 4 = 1-x, 2-y, 1 – z.

Table S6. Bond angles for $[Dy_2(C_6O_4Cl_2)_3(\mu\text{-eg-k}O)(H_2O)_4]\cdot2eg\cdot7H_2O$ (**3**).

Atom	Atom	Atom	Angle (˚)	Atom	Atom	Atom	Angle (˚)	Atom	Atom	Atom	Angle (˚)
O22	Dy1	O12W	69.62(13)	$O26^2$	Dy1	$O16^1$	125.49(15)	O26	C26	$C22^2$	114.6(6)
O22	Dy1	$O16^1$	137.06(14)	O1E	Dy1	O12W	127.74(15)	O26	C26	C21	124.3(6)
O22	Dy1	$O26^2$	65.30(14)	O1E	Dy1	$O16^1$	73.95(17)	C21	C26	$C22^2$	121.1(5)
O22	Dy1	O1E	70.77(16)	O1E	Dy1	$O26^2$	73.28(18)	C16	C11	Cl11	118.2(5)
O12	Dy1	O12W	69.28(13)	O11W	Dy1	O12W	145.13(15)	C12	C11	Cl11	119.6(5)
O12	Dy1	O22	81.54(14)	O11W	Dy1	O22	131.31(16)	C12	C11	C16	122.1(6)
O12	Dy1	$O16^1$	64.88(13)	O11W	Dy1	O12	133.07(15)	O6	C6	$C2^3$	114.1(5)
O12	Dy1	$O26^2$	138.35(14)	O11W	Dy1	$O6^3$	79.54(16)	O6	C6	C1	126.9(6)
O12	Dy1	O1E	72.41(17)	O11W	Dy1	$O16^1$	69.05(16)	C1	C6	$C2^3$	118.9(6)
$O6^3$	Dy1	O12W	70.44(14)	O11W	Dy1	$O26^2$	66.96(16)	O16	C16	C11	125.2(6)
$O6^3$	Dy1	O22	94.04(14)	O11W	Dy1	O1E	87.10(17)	O16	C16	$C12^1$	115.6(5)
$O6^3$	Dy1	O12	138.39(14)	C22	O22	Dy1	121.6(4)	C11	C16	$C12^1$	119.2(6)
$O6^3$	Dy1	$O16^1$	128.79(14)	C12	O12	Dy1	122.5(4)	C22	C21	Cl21	119.2(5)
$O6^3$	Dy1	$O26^2$	71.49(15)	C6	O6	$Dy1^3$	119.7(4)	C26	C21	Cl21	119.8(5)
$O6^3$	Dy1	O1E	144.77(19)	C16	O16	$Dy1^1$	121.5(4)	C26	C21	C22	120.9(5)
$O16^1$	Dy1	O12W	117.41(15)	C2	O2	Dy1	121.2(4)	O12	C12	C11	125.8(6)
O2	Dy1	O12W	73.28(13)	C26	O26	$Dy1^2$	121.6(4)	O12	C12	$C16^1$	115.5(6)
O2	Dy1	O22	142.20(13)	C1E	O1E	Dy1	128.5(5)	C11	C12	$C16^1$	118.7(5)
O2	Dy1	O12	92.17(15)	O2	C2	$C6^3$	116.2(5)	C2	C1	Cl1	117.6(5)
O2	Dy1	$O6^3$	66.46(15)	O2	C2	C1	125.1(6)	C6	C1	Cl1	120.1(5)
O2	Dy1	$O16^1$	68.45(14)	C1	C2	$C6^3$	118.7(5)	C6	C1	C2	122.3(6)
O2	Dy1	$O26^2$	129.48(14)	O22	C22	$C26^2$	116.0(5)	O102	C102	C101	111.2(11)
O2	Dy1	O1E	142.38(17)	O22	C22	C21	126.2(6)	O101	C101	C102	112.2(10)
O2	Dy1	O11W	78.83(16)	C21	C22	$C26^2$	117.8(6)	$C1E^4$	C1E	O1E	104.2(12)
$O26^2$	Dy1	O12W	117.10(14)								

1 = 1-x, 1-y, 1-z; 2 = 2-x, 2-y, 1-z ; 3 = 2-x, 1-y, -z; 4 = 1-x, 2-y, 1 – z.

Table S7. Bond distances for $[Dy_2(C_6O_4Br_2)_3(eg\text{-}k^2O,O')_2(CH_3OH)_2]\cdot 2eg\cdot 4CH_3OH$ (**4**).

Atom	Atom	Distance (Å)	Atom	Atom	Distance (Å)	Atom	Atom	Distance (Å)
Dy1	O12	2.397(10)	O12	C12	1.246(18)	C1	C2	1.41(2)
Dy1	$O6^1$	2.422(11)	O6	C6	1.257(19)	C4	C3	1.35(2)
Dy1	O3	2.369(9)	O3	C3	1.263(18)	C11	C12	1.38(2)
Dy1	$O5^1$	2.350(11)	O5	C5	1.284(18)	C11	C16	1.37(2)
Dy1	$O16^2$	2.461(10)	O16	C16	1.256(18)	C12	$C16^2$	1.56(2)
Dy1	O2	2.454(10)	O2	C2	1.237(18)	O102	C102	1.46(2)
Dy1	O11E	2.412(10)	O11E	C11E	1.463(16)	C3	C2	1.56(2)
Dy1	O11M	2.404(12)	O11M	C11M	1.42(2)	O101	C101	1.45(2)
Dy1	O12E	2.425(9)	O12E	C12E	1.473(16)	C12E	C11E	1.46(2)
Br11	C11	1.896(14)	C5	C4	1.42(2)	C101	C102	1.44(3)
Br4	C4	1.878(16)	C5	C6	1.54(2)	O1M	C1M	1.38(3)
Br1	C1	1.865(16)	C1	C6	1.39(2)	O2M	C2M	1.46(3)

1 = x, 3/2-y, ½ + z; 2 = 1-x, 1-y, 1-z.

Table S8. Bond angles for $[Dy_2(C_6O_4Br_2)_3(eg\text{-}k^2O,O')_2(CH_3OH)_2]\cdot 2eg\cdot 4CH_3OH$ (**4**).

Atom	Atom	Atom	Angle (°)	Atom	Atom	Atom	Angle (°)	Atom	Atom	Atom	Angle (°)
O12	Dy1	$O6^1$	136.8(4)	O11E	Dy1	$O16^2$	68.9(4)	C3	C4	Br4	120.9(12)
O12	Dy1	$O16^2$	64.3(3)	O11E	Dy1	O2	68.8(3)	C3	C4	C5	121.6(15)
O12	Dy1	O2	67.2(4)	O11E	Dy1	O12E	66.7(4)	C12	C11	Br11	117.6(11)
O12	Dy1	O11E	87.1(4)	O11M	Dy1	$O6^1$	133.8(4)	C16	C11	Br11	120.1(11)
O12	Dy1	O11M	77.1(4)	O11M	Dy1	$O16^2$	68.0(4)	C16	C11	C12	122.1(14)
O12	Dy1	O12E	136.9(3)	O11M	Dy1	O2	135.1(4)	O12	C12	C11	126.4(14)
$O6^1$	Dy1	$O16^2$	145.9(4)	O11M	Dy1	O11E	136.8(4)	O12	C12	$C16^2$	113.1(13)
$O6^1$	Dy1	O2	70.2(4)	O11M	Dy1	O12E	98.7(4)	C11	C12	$C16^2$	120.5(14)
$O6^1$	Dy1	O12E	76.1(4)	O12E	Dy1	$O16^2$	74.3(4)	O6	C6	C5	113.8(13)
O3	Dy1	O12	72.3(3)	O12E	Dy1	O2	125.9(4)	O6	C6	C1	127.2(16)
O3	Dy1	$O6^1$	84.1(4)	C12	O12	Dy1	122.7(10)	C1	C6	C5	118.9(14)
O3	Dy1	$O16^2$	129.6(4)	C6	O6	$Dy1^3$	121.0(10)	O3	C3	C4	126.0(15)
O3	Dy1	O2	65.3(4)	C3	O3	Dy1	118.3(9)	O3	C3	C2	113.8(13)
O3	Dy1	O11E	134.0(4)	C5	O5	$Dy1^3$	122.0(10)	C4	C3	C2	120.1(14)
O3	Dy1	O11M	78.8(4)	C16	O16	$Dy1^2$	118.3(10)	O2	C2	C1	126.5(15)
O3	Dy1	O12E	149.9(4)	C2	O2	Dy1	117.4(10)	O2	C2	C3	115.3(13)
$O5^1$	Dy1	O12	138.6(4)	C11E	O11E	Dy1	116.9(9)	C1	C2	C3	118.1(13)
$O5^1$	Dy1	$O6^1$	65.8(4)	C11M	O11M	Dy1	128.3(12)	O16	C16	C11	127.5(14)
$O5^1$	Dy1	O3	78.6(4)	C12E	O12E	Dy1	120.0(9)	O16	C16	$C12^2$	115.2(13)
$O5^1$	Dy1	$O16^2$	119.6(4)	O5	C5	C4	125.9(14)	C11	C16	$C12^2$	117.3(13)
$O5^1$	Dy1	O2	124.9(4)	O5	C5	C6	115.0(13)	C11E	C12E	O12E	107.9(12)
$O5^1$	Dy1	O11E	134.1(4)	C4	C5	C6	119.1(13)	C12E	C11E	O11E	111.1(13)
$O5^1$	Dy1	O11M	68.8(4)	C6	C1	Br1	118.4(12)	C102	C101	O101	111.4(17)
$O5^1$	Dy1	O12E	72.7(4)	C6	C1	C2	121.8(15)	C101	C102	O102	107.9(17)
O2	Dy1	$O16^2$	115.5(4)	C2	C1	Br1	119.7(12)				
O11E	Dy1	$O6^1$	84.0(4)	C5	C4	Br4	117.5(11)				

1 = x, 3/2-y, ½ + z; 2 = 1-x, 1-y, 1-z; 3 = x, 3/2-y, -1/2 + z.

Table S9. Bond distances for $[Dy_2(C_6O_4Br_2)_3(eg\text{-}k^2\mathit{O,O'})_2(eg\text{-}k\mathit{O})_2]\cdot 4eg$ (**5**).

Atom	Atom	Distance (Å)	Atom	Atom	Distance (Å)	Atom	Atom	Distance (Å)
Dy1	O16^{1}	2.489(6)	O26	C26	1.253(10)	C6	C1	1.374(12)
Dy1	O26^{2}	2.436(6)	O2	C2	1.259(11)	C21	C22	1.383(12)
Dy1	O2	2.355(6)	O6	C6	1.258(10)	C21	C26	1.391(12)
Dy1	O6^{3}	2.359(6)	O11E	C11E	1.444(10)	C12	C16^{1}	1.526(12)
Dy1	O11E	2.374(7)	O22	C22	1.247(10)	C12	C11	1.379(12)
Dy1	O22	2.380(6)	O1E	C1E	1.436(10)	O201	C201	1.408(14)
Dy1	O1E	2.408(5)	O12E	C12E	1.444(12)	C22	C26^{2}	1.543(12)
Dy1	O12E	2.464(6)	O12	C12	1.274(11)	C16	C11	1.405(13)
Dy1	O12	2.377(6)	O202	C202	1.410(8)	O2E	C2E	1.442(13)
Br1	C1	1.900(8)	O102	C102	1.401(12)	C12E	C11E	1.506(13)
Br11	C11	1.889(9)	O101	C101	1.430(11)	C202	C201	1.478(14)
Br21	C21	1.896(9)	C2	C6^{3}	1.539(11)	C1E	C2E	1.500(14)
O16	C16	1.245(10)	C2	C1	1.392(13)	C102	C101	1.482(14)

1 = 1-x, 1-y, 2-z; 2 = 1-x, 2-y, 1-z; 3 = 1-x, 2-y, 2 – z.

Table S10. Bond angles for $[Dy_2(C_6O_4Br_2)_3(eg\text{-}k^2\mathit{O,O'})_2(eg\text{-}k\mathit{O})_2]\cdot 4eg$ (**5**).

Atom	Atom	Atom	Angle (°)	Atom	Atom	Atom	Angle (°)	Atom	Atom	Atom	Angle (°)
O26^{1}	Dy1	O16^{2}	138.4(2)	O1E	Dy1	O16^{2}	70.1(2)	O12	C12	C16^{2}	113.6(7)
O26^{1}	Dy1	O12E	72.7(2)	O1E	Dy1	O26^{1}	134.0(2)	O12	C12	C11	126.9(9)
O2	Dy1	O16^{2}	70.3(2)	O1E	Dy1	O12E	140.3(2)	C11	C12	C16^{2}	119.5(8)
O2	Dy1	O26^{1}	76.5(2)	O12E	Dy1	O16^{2}	111.4(2)	O22	C22	C21	125.3(8)
O2	Dy1	O6^{3}	67.2(2)	O12	Dy1	O16^{2}	64.0(2)	O22	C22	C26^{1}	115.8(8)
O2	Dy1	O11E	147.4(2)	O12	Dy1	O26^{1}	144.6(2)	C21	C22	C26^{1}	118.9(8)
O2	Dy1	O22	73.6(2)	O12	Dy1	O22	131.2(2)	O16	C16	C12^{2}	115.8(8)
O2	Dy1	O1E	88.5(2)	O12	Dy1	O1E	73.1(2)	O16	C16	C11	124.6(9)
O2	Dy1	O12E	130.4(2)	O12	Dy1	O12E	72.9(2)	C11	C16	C12^{2}	119.5(8)
O2	Dy1	O12	134.1(2)	C16	O16	Dy1^{2}	118.8(6)	O26	C26	C21	126.6(9)
O6^{3}	Dy1	O16^{2}	67.9(2)	C26	O26	Dy1^{1}	119.5(6)	O26	C26	C22^{1}	115.2(8)
O6^{3}	Dy1	O26^{1}	76.5(2)	C2	O2	Dy1	118.1(5)	C21	C26	C22^{1}	118.3(8)
O6^{3}	Dy1	O11E	134.7(2)	C6	O6	Dy1^{3}	117.4(5)	C12	C11	Br11	118.9(7)
O6^{3}	Dy1	O22	130.6(2)	C11E	O11E	Dy1	118.3(5)	C12	C11	C16	121.0(9)
O6^{3}	Dy1	O1E	136.5(2)	C22	O22	Dy1	121.5(5)	C16	C11	Br11	120.1(7)
O6^{3}	Dy1	O12E	68.5(2)	C1E	O1E	Dy1	134.4(6)	O12E	C12E	C11E	107.3(8)
O6^{3}	Dy1	O12	97.9(2)	C12E	O12E	Dy1	117.9(5)	C2	C1	Br1	118.0(7)
O11E	Dy1	O16^{2}	135.3(2)	C12	O12	Dy1	123.2(6)	C6	C1	Br1	118.9(7)
O11E	Dy1	O26^{1}	85.5(2)	O2	C2	C6^{3}	114.5(8)	C6	C1	C2	123.1(8)
O11E	Dy1	O22	74.2(2)	O2	C2	C1	127.1(8)	O11E	C11E	C12E	108.2(8)
O11E	Dy1	O1E	84.7(2)	C1	C2	C6^{3}	118.3(8)	O202	C202	C201	115.3(9)
O11E	Dy1	O12E	66.5(2)	O6	C6	C2^{3}	115.7(8)	O1E	C1E	C2E	113.1(8)
O11E	Dy1	O12	73.8(2)	O6	C6	C1	125.7(8)	O2E	C2E	C1E	112.0(9)
O22	Dy1	O16^{2}	124.6(2)	C1	C6	C2^{3}	118.5(8)	O102	C102	C101	110.1(9)
O22	Dy1	O26^{1}	65.86(19)	C22	C21	Br21	119.1(7)	O101	C101	C102	112.2(8)
O22	Dy1	O1E	68.3(2)	C22	C21	C26	122.9(8)	O201	C201	C202	111.0(10)
O22	Dy1	O12E	124.0(2)	C26	C21	Br21	118.1(7)				

1 = 1-x, 2-y, 1-z; 2 = 1-x, 1-y, 2-z; 3 = 1-x, 2-y, 2-z.

Table S11. Bond distances for $[Dy_2(C_6O_4Br_2)_3(eg\text{-}k^2O,O')_2(eg\text{-}kO)(H_2O)]\cdot 2eg\cdot H_2O$ (**6**).

Atom	Atom	Distance (Å)	Atom	Atom	Distance (Å)	Atom	Atom	Distance (Å)
Dy2	O22E	2.404(4)	Br11	C11	1.910(7)	C25	C26	1.537(8)
Dy2	O5	2.421(4)	O11E	C11E	1.437(7)	C23	C22	1.545(8)
Dy2	O6	2.363(4)	O22E	C22E	1.424(7)	C23	C24	1.388(9)
Dy2	O23	2.359(4)	O11	C12	1.255(7)	C21	C22	1.384(9)
Dy2	O21E	2.418(4)	O5	C5	1.263(8)	C21	C26	1.395(9)
Dy2	$O36^1$	2.424(4)	O6	C6	1.250(8)	O2E	C2E	1.445(11)
Dy2	O22	2.387(4)	O23	C23	1.261(8)	C12	$C16^3$	1.540(9)
Dy2	O32	2.404(4)	O21E	C21E	1.433(7)	C12	C11	1.372(9)
Dy2	O11W	2.477(4)	O36	C36	1.264(7)	C2	C3	1.522(8)
Dy1	O11E	2.397(4)	O2	C2	1.277(8)	C6	C5	1.545(8)
Dy1	O11	2.381(4)	O1E	C1E	1.455(8)	C16	C11	1.402(8)
Dy1	O2	2.496(4)	O3	C3	1.260(8)	C3	C4	1.391(9)
Dy1	O1E	2.442(4)	O26	C26	1.245(8)	C32	C31	1.386(9)
Dy1	O3	2.356(4)	O22	C22	1.258(8)	C32	$C36^1$	1.546(9)
Dy1	$O26^2$	2.400(5)	O16	C16	1.239(8)	C4	C5	1.380(9)
Dy1	$O16^3$	2.392(4)	O12E	C12E	1.444(7)	C22E	C21E	1.489(9)
Dy1	O12E	2.390(4)	O32	C32	1.258(7)	C11E	C12E	1.496(8)
Dy1	$O25^2$	2.434(4)	O25	C25	1.258(8)	C31	C36	1.384(8)
Br1	C1	1.887(6)	O201	C201	1.415(8)	O102	C102	1.433(9)
Br24	C24	1.885(6)	O202	C202	1.435(8)	O101	C101	1.429(9)
Br4	C4	1.879(6)	C1	C2	1.366(10)	C1E	C2E	1.501(11)
Br31	C31	1.889(6)	C1	C6	1.392(9)	C201	C202	1.495(10)
Br21	C21	1.886(6)	C25	C24	1.378(9)	C102	C101	1.487(11)

1 = 1-x, 1-y, -z; 2 * -1+x, 1+y, +z; 3 = -x, 2-y, 1-z.

Table S12. Bond angles for $[Dy_2(C_6O_4Br_2)_3(eg\text{-}k^2\mathit{O,O'})_2(eg\text{-}k\mathit{O})(H_2O)]\cdot 2eg\cdot H_2O$ (**6**).

Atom	Atom	Atom	Angle (˚)	Atom	Atom	Atom	Angle (˚)	Atom	Atom	Atom	Angle (˚)
O22E	Dy2	O5	70.75(14)	O3	Dy1	O1E	67.05(15)	O22	C22	C21	126.7(6)
O22E	Dy2	O21E	65.63(14)	O3	Dy1	O26^{2}	138.65(14)	C21	C22	C23	119.0(6)
O22E	Dy2	O36^{1}	116.77(13)	O3	Dy1	O16^{3}	69.45(15)	O11	C12	C16^{3}	114.8(6)
O22E	Dy2	O11W	126.77(14)	O3	Dy1	O12E	78.70(15)	O11	C12	C11	126.9(6)
O5	Dy2	O36^{1}	72.99(14)	O3	Dy1	O25^{2}	110.83(14)	C11	C12	C16^{3}	118.3(5)
O5	Dy2	O11W	120.76(16)	O26^{2}	Dy1	O2	140.53(13)	O2	C2	C1	126.9(6)
O6	Dy2	O22E	131.31(15)	O26^{2}	Dy1	O1E	73.85(15)	O2	C2	C3	114.2(6)
O6	Dy2	O5	65.79(14)	O26^{2}	Dy1	O25^{2}	65.17(14)	C1	C2	C3	118.9(6)
O6	Dy2	O21E	82.36(15)	O16^{3}	Dy1	O11E	124.41(13)	O6	C6	C1	126.4(6)
O6	Dy2	O36^{1}	70.01(14)	O16^{3}	Dy1	O2	76.73(14)	O6	C6	C5	114.3(6)
O6	Dy2	O22	103.16(14)	O16^{3}	Dy1	O1E	104.01(14)	C1	C6	C5	119.2(6)
O6	Dy2	O32	134.60(14)	O16^{3}	Dy1	O26^{2}	135.29(16)	O16	C16	C12^{3}	114.8(5)
O6	Dy2	O11W	64.62(15)	O16^{3}	Dy1	O25^{2}	72.28(15)	O16	C16	C11	126.6(6)
O23	Dy2	O22E	73.17(14)	O12E	Dy1	O11E	66.41(14)	C11	C16	C12^{3}	118.6(6)
O23	Dy2	O5	142.03(13)	O12E	Dy1	O2	77.96(14)	C25	C24	Br24	118.1(5)
O23	Dy2	O6	138.03(14)	O12E	Dy1	O1E	74.18(15)	C25	C24	C23	121.4(6)
O23	Dy2	O21E	80.08(15)	O12E	Dy1	O26^{2}	78.25(16)	C23	C24	Br24	120.5(5)
O23	Dy2	O36^{1}	136.05(15)	O12E	Dy1	O16^{3}	145.49(16)	O3	C3	C2	115.8(6)
O23	Dy2	O22	66.51(14)	O12E	Dy1	O25^{2}	133.90(15)	O3	C3	C4	124.3(6)
O23	Dy2	O32	83.04(15)	O25^{2}	Dy1	O2	147.78(14)	C4	C3	C2	119.9(6)
O23	Dy2	O11W	73.50(15)	O25^{2}	Dy1	O1E	69.54(16)	O26	C26	C25	115.9(6)
O21E	Dy2	O5	74.65(15)	C11E	O11E	Dy1	116.6(3)	O26	C26	C21	125.3(6)
O21E	Dy2	O36^{1}	143.85(16)	C22E	O22E	Dy2	118.9(3)	C21	C26	C25	118.8(6)
O21E	Dy2	O11W	68.66(14)	C12	O11	Dy1	119.7(4)	O32	C32	C31	126.4(6)
O36^{1}	Dy2	O11W	116.12(13)	C5	O5	Dy2	120.7(4)	O32	C32	C36^{1}	114.2(6)
O22	Dy2	O22E	125.46(15)	C6	O6	Dy2	123.9(4)	C31	C32	C36^{1}	119.3(5)
O22	Dy2	O5	148.02(14)	C23	O23	Dy2	121.7(4)	C3	C4	Br4	119.0(5)
O22	Dy2	O21E	135.68(14)	C21E	O21E	Dy2	118.0(3)	C5	C4	Br4	119.7(5)
O22	Dy2	O36^{1}	75.03(14)	C36	O36	Dy2^{1}	121.9(4)	C5	C4	C3	121.1(6)
O22	Dy2	O32	72.27(14)	C2	O2	Dy1	119.8(4)	O5	C5	C6	115.3(6)
O22	Dy2	O11W	74.31(15)	C1E	O1E	Dy1	131.8(4)	O5	C5	C4	126.3(6)
O32	Dy2	O22E	67.71(13)	C3	O3	Dy1	124.4(4)	C4	C5	C6	118.4(6)
O32	Dy2	O5	93.93(15)	C26	O26	Dy1^{4}	122.2(4)	O22E	C22E	C21E	105.5(5)
O32	Dy2	O21E	133.16(13)	C22	O22	Dy2	121.4(4)	O11E	C11E	C12E	105.6(5)
O32	Dy2	O36^{1}	65.17(14)	C16	O16	Dy1^{3}	120.3(4)	C32	C31	Br31	119.9(5)
O32	Dy2	O11W	144.72(16)	C12E	O12E	Dy1	118.2(3)	C36	C31	Br31	118.5(5)
O11E	Dy1	O2	70.15(14)	C32	O32	Dy2	123.3(4)	C36	C31	C32	121.4(6)
O11E	Dy1	O1E	131.58(14)	C25	O25	Dy1^{4}	121.1(4)	C12	C11	Br11	119.1(5)
O11E	Dy1	O26^{2}	71.61(14)	C2	C1	Br1	120.2(5)	C12	C11	C16	123.1(6)
O11E	Dy1	O25^{2}	121.79(15)	C2	C1	C6	121.8(6)	C16	C11	Br11	117.8(5)
O11	Dy1	O11E	70.24(14)	C6	C1	Br1	117.8(5)	O36	C36	C32^{1}	115.0(5)
O11	Dy1	O2	88.78(15)	O25	C25	C24	125.6(6)	O36	C36	C31	126.0(6)
O11	Dy1	O1E	140.14(16)	O25	C25	C26	114.5(6)	C31	C36	C32^{1}	119.0(5)
O11	Dy1	O26^{2}	87.35(15)	C24	C25	C26	119.8(6)	O21E	C21E	C22E	108.4(5)
O11	Dy1	O16^{3}	65.63(14)	O23	C23	C22	115.3(6)	O12E	C12E	C11E	106.1(5)
O11	Dy1	O12E	136.63(13)	O23	C23	C24	125.4(6)	O1E	C1E	C2E	113.5(7)
O11	Dy1	O25^{2}	70.69(15)	C24	C23	C22	119.3(6)	O201	C201	C202	114.1(7)
O1E	Dy1	O2	127.81(15)	C22	C21	Br21	119.0(5)	O202	C202	C201	110.9(6)
O3	Dy1	O11E	127.37(15)	C22	C21	C26	121.6(6)	O2E	C2E	C1E	111.8(8)
O3	Dy1	O11	131.94(14)	C26	C21	Br21	119.2(5)	O102	C102	C101	113.2(7)
O3	Dy1	O2	64.76(13)	O22	C22	C23	114.3(6)	O101	C101	C102	109.2(7)

1 = 1-x, 1-y, -z; 2 = -1+x, 1+y, +z; 3 = -x, 2-y, 1-z; 4 = 1+x, -1+y, +z.

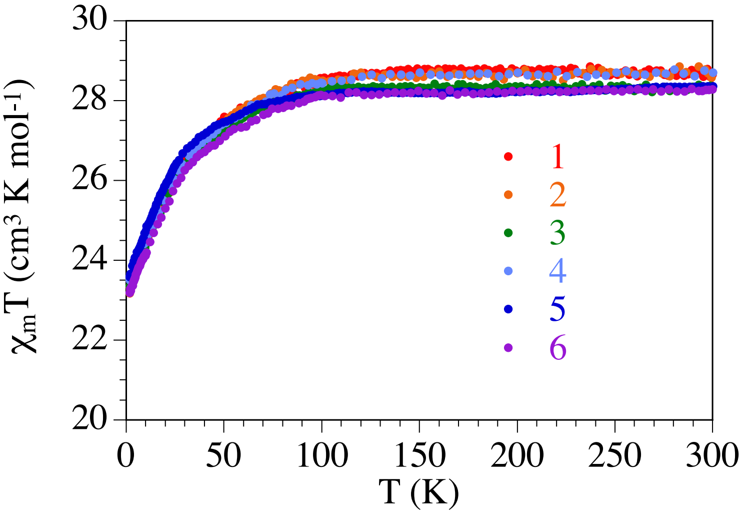

***Figure S7**. Thermal variation of the $\chi_m T$ product for compounds **1-6** per formula unit (two Dy(III) ions).*

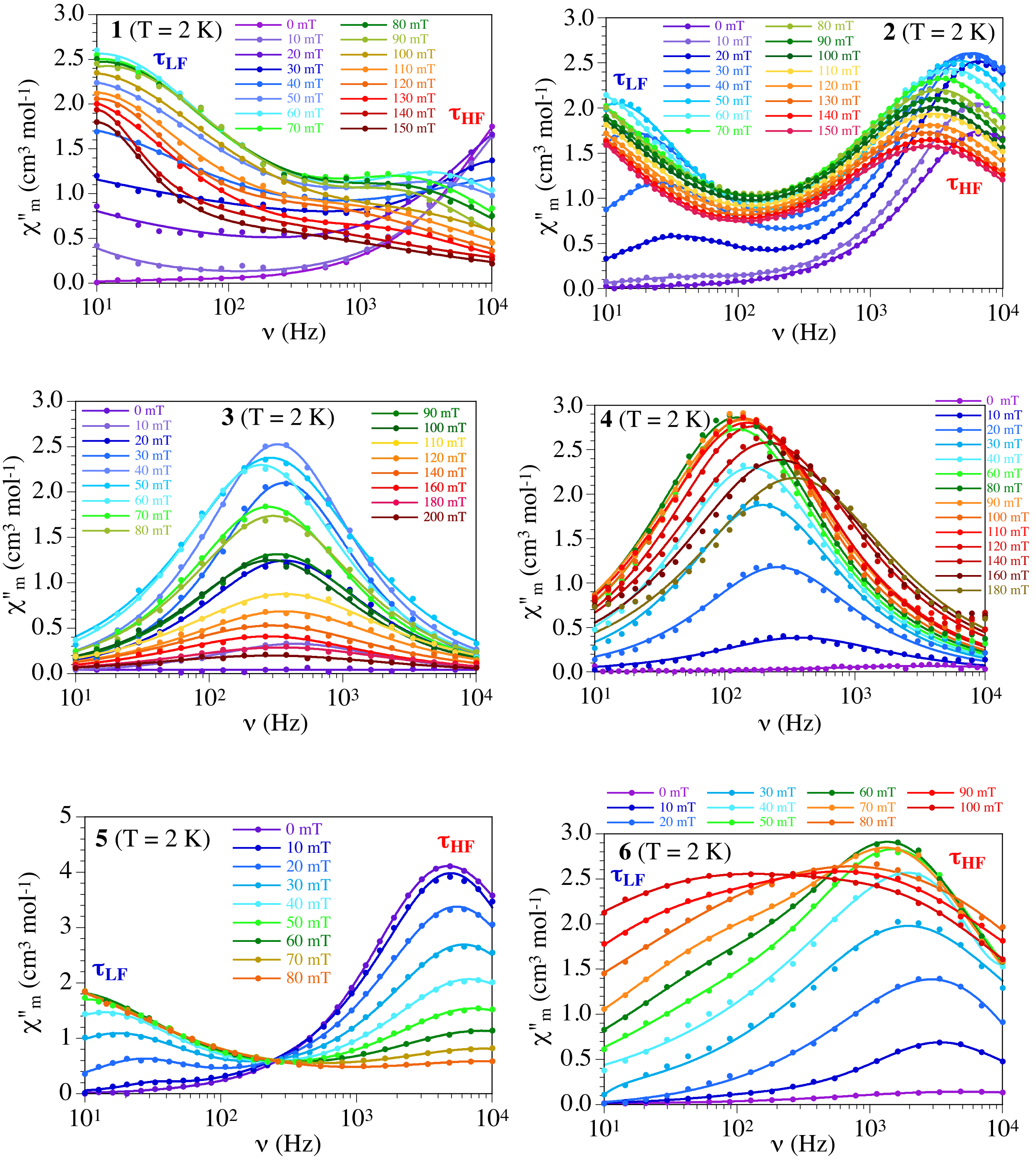

Figure S8*. Frequency dependence of the χ"m signal at 2 K with different applied DC fields for compounds* ***1*****-*****6****. Solid lines are the fits to the Debye model with one (*****3*** *and* ***4****) or two (*****1*****,* ***2*****,* ***5*** *and* ***6****) relaxation processes.*

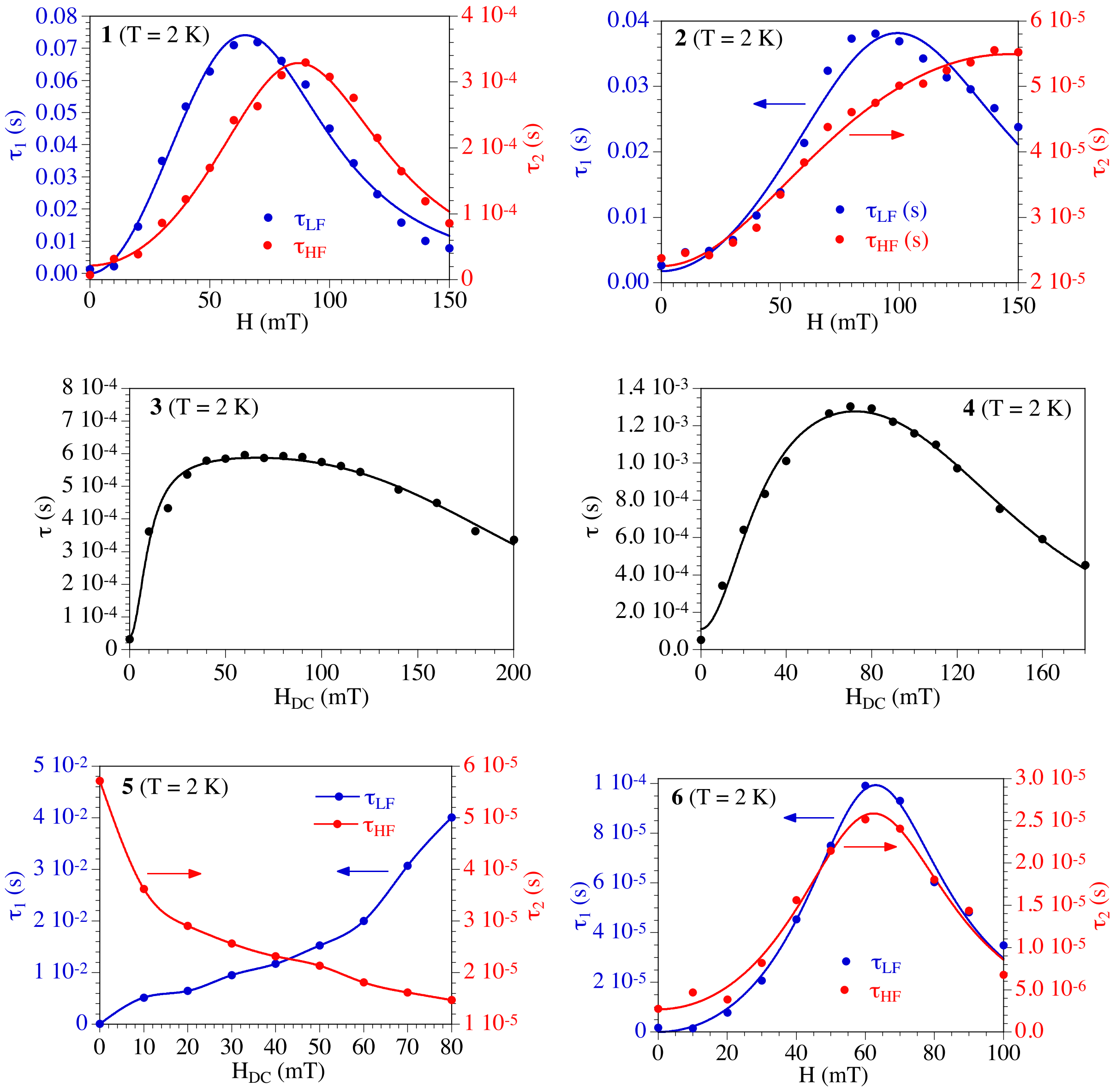

***Figure S9**. Field dependence of the relaxation times at 2 K for compounds **1**-**6**. Solid lines are the best fits to equation (1) (except in compound **5**) with the parameters displayed in Table S13.*

The field dependence of the relaxation time can be fitted to equation (1) with n = 4 for Kramers ions as Dy(III).[1]

$$\tau^{-1} = AH^n + \frac{B_1}{1+B_2H^2} + D \qquad (1)$$

Table S13. Magnetic parameters obtained with equation (1) with n = 4, for compounds **1-6** at T = 2.0 K.

Compound	process	A (s^{-1} mT^{-4})	B_1 (s^{-1})	B_2 (mT^{-2})	D (s^{-1})
**1**	τ_{LF}	1.3(2)x10^{-10}	6.6(5)x10^{2}	3.6(3)x10^{-2}	8.4(6)
	τ_{HF}	1.8(1)x10^{-5}	4.7(2)x10^{4}	2.7(1)x10^{-3}	1.5(3)x10^{2}
**2**	τ_{LF}	7(1)x10^{-8}	5.6(5)x10^{2}	3.8(4)x10^{-3}	5.1(4)
	τ_{HF}	3.2(2)x10^{-6}	3.1(2)x10^{4}	3.9(8)x10^{-4}	1.3(1)x10^{4}
**3**	τ_{HF}	1.5(2)x10^{-6}	6.5(1)x10^{4}	2.2(4)x10^{-1}	1.9(1)x10^{3}
**4**	τ_{HF}	1.9(4)x10^{-6}	8.4(3)x10^{3}	1.8(7)x10^{-2}	6.5(3)x10^{2}
**6**	τ_{LF}	4.2(1)x10^{-1}	1.1(3)x10^{6}	1.8(4)x10^{-2}	1.9(1)x10^{4}
	τ_{HF}	1.2(1)x10^{-3}	3.9(1)x10^{5}	2.2(1)x10^{-3}	2.0(1)x10^{4}

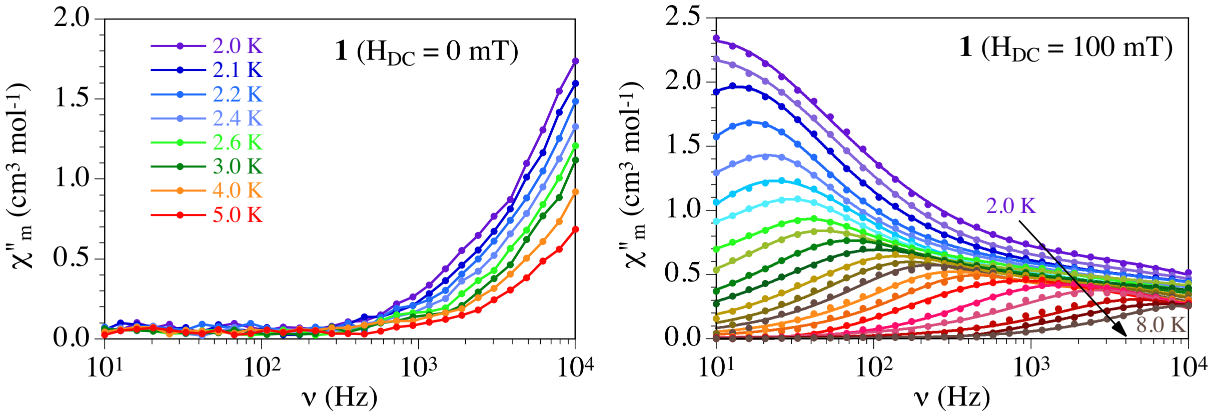

***Figure S10**. Frequency dependence of χ''_m for compound **1** at different temperatures with H_{DC} = 0 (left) and 100 mT (right). Solid line for H_{DC} = 100 mT is the best fit to a Debye model with two relaxation processes.*

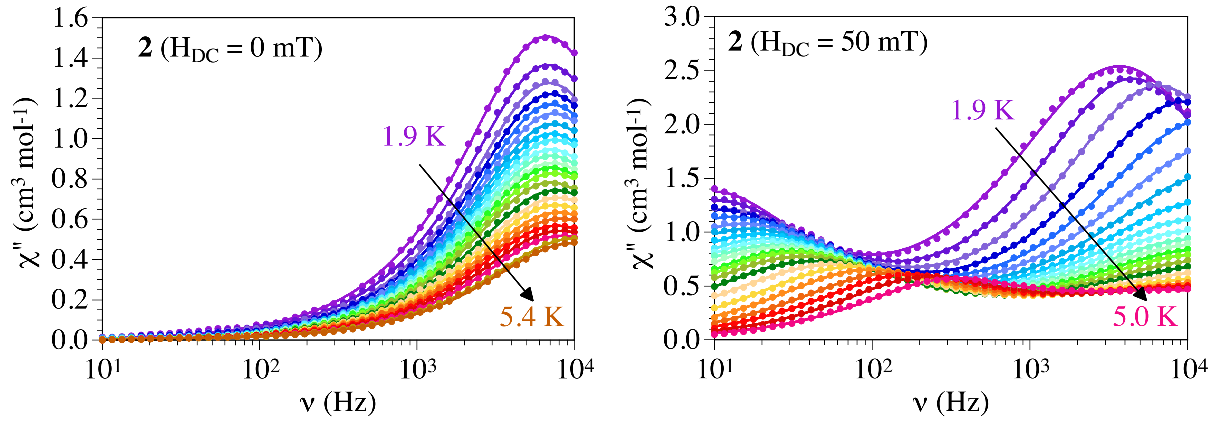

***Figure S11**. Frequency dependence of χ''_m for compound **2** at different temperatures with H_{DC} = 0 (left) and 50 mT (right). Solid lines are the best fit to a Debye model with one (0 mT) or two (50 mT) relaxation processes.*

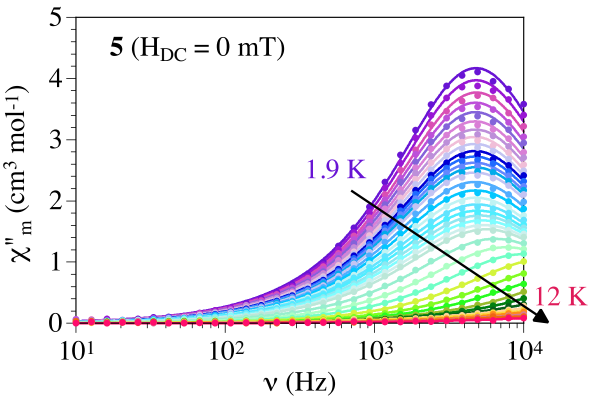

Figure S12. *Frequency dependence of χ''$_m$ for compound* ***5*** *at different temperatures with H_{DC} = 0. Solid lines are the best fit to a Debye model with one relaxation processes.*

References

(1) Dey, A.; Kalita, P.; Chandrasekhar, V. Lanthanide(III)-Based Single-Ion Magnets. ACS Omega **2018**, *3*, 9462-9475.
